# Evolution in Bone Tissue Regeneration: From Grafts to Innovative Biomaterials

**DOI:** 10.3390/ijms26094242

**Published:** 2025-04-29

**Authors:** Domingo Cesar Carrascal-Hernández, Juan Pablo Martínez-Cano, Juan David Rodríguez Macías, Carlos David Grande-Tovar

**Affiliations:** 1 Departamento de Química y Biología, Facultad de Ciencias Básicas, Universidad del Norte, Barranquilla 080020, Colombia; domingoh@uninorte.edu.co; 2 Ortopedia y Traumatología, Epidemiología Clínica, Fundación Valle del Lili, Universidad ICESI, Cali 760031, Colombia; juan.martinez.ca@fvl.org.co; 3 Programa de Medicina, Facultad de Ciencias de la Salud, Universidad Libre, Barranquilla 081007, Colombia; juand.rodriguezm@unilibre.edu.co; 4 Grupo de Investigación en Fotoquímica y Fotobiología, Programa de Química, Universidad del Atlántico, Puerto Colombia 081007, Colombia

**Keywords:** biomaterials, bone regeneration, mesenchymal stem cells, smart biomaterials

## Abstract

Bone defects caused by various traumas and diseases such as osteoporosis, which affects bone density, and osteosarcoma, which affects the integrity of bone structure, are now well known. Given this situation, several innovative research projects have been reported to improve orthopedic methods and technologies that positively contribute to the regeneration of affected bone tissue, representing a significant advance in regenerative medicine. This review article comprehensively analyzes the transition from existing methods and technologies for implants and bone tissue regeneration to innovative biomaterials. These biomaterials have been of great interest in the last decade due to their physicochemical characteristics, which allow them to overcome the most common limitations of traditional grafting methods, such as the availability of biomaterials and the risk of rejection after their application in regenerative medicine. This could be achieved through an exhaustive study of the applications and properties of various materials with potential applications in regenerative medicine, such as using magnetic nanoparticles and hydrogels sensitive to external stimuli, including pH and temperature. In this regard, this review article describes the most relevant compounds used in bone tissue regeneration, promoting the integration of these biomaterials with the affected area’s bone structure, thereby allowing for regeneration and preventing amputation. Additionally, the types of interactions between biomaterials and mesenchymal stem cells and their effects on bone tissue are discussed, which is critical for developing biomaterials with optimal regenerative properties. Furthermore, the mechanisms of action of the various biomaterials that enhance osteoconduction and osteoinduction, ensuring the success of orthopedic therapies, are analyzed. This enables the treatment of bone defects tailored to each patient’s condition, thereby avoiding limb amputation. Consequently, a promising future for regenerative medicine is emerging, with various therapies that could revolutionize the management of bone defects, offering more efficient and safer solutions.

## 1. Introduction

Bone tissue comprises a highly dynamic and complex system, which is essential because it provides structural support and protects vital organs, among other functions. This complex system is dynamic because it involves the storage of various crucial minerals for the body and regulating chemical balance, which is vital for repairing bone tissue after injuries caused by trauma or disease [1]. This process is slow, even with the repair efficiency of these dynamic systems that promote bone regeneration. The new bone tissue has low mechanical resistance, resulting in physical limitations for patients suffering from serious injuries [2,3].

In this context, various traumas, surgical failures, and diseases that compromise the integrity of bone tissue have been reported. However, multiple bone grafts and osteogenic biomaterials have been used in recent applications to accelerate bone tissue regeneration while preserving the quality and quantity of bone mass [4]. Unfortunately, traditional bone graft applications face several challenges. For example, they exhibit poor integration with bone tissue, which represents a high risk of immune rejection; they are often scarce, resulting in low availability; and they cannot also heal, hindering the migration of cells and minerals essential for regeneration [5]. Given this situation, there is urgent need to implement new porous materials that support the affected area and promote the migration of cells and minerals, thereby preventing unfavorable healing of bone tissue and avoiding the risk of heterotropic pseudoarthrosis [6].

Various bone implants have recently been reported to address and overcome these limitations. For example, autografts, xenografts, allografts, and various scaffolds are based on multiple biomaterials [7]. In this sense, autologous tissue extracted from the patient’s body yields the best results, possessing excellent osteogenic, osteoinductive, and osteoconductive properties, minimizing the risk of immune rejection [8]. The most suitable extraction sites are usually the ileum, fibula, and ribs [9,10,11]. The mechanism of bone regeneration involves the production of new intramembranous bone cells, where MSCs play a crucial role through a differentiation process that generates osteoblasts. Bioactive factors such as bone morphogenetic proteins (BMPs) promote differentiation, facilitating tissue regeneration [12,13].

Furthermore, autografts have limitations that make their application difficult. For example, their application requires additional surgical procedures on the patient and limited tissue availability [11]. To overcome these limitations, the application of allografts has been reported; allografts are obtained from donors of the same species, which increases their availability. That is why they have been used as scaffolds to promote osteoconduction, the migration of cells and minerals that induce bone regeneration, and provide support and rigidity in the affected area [14]. This material has been applied as freeze-dried bone/tissue and as a demineralized bone matrix [15]. However, it also has significant limitations, including a high risk of immune rejection and disease transmission.

As an emerging alternative, synthetic bone materials have gained interest due to their effectiveness in bone regeneration [16,17,18,19,20]. These include metals [21], bioceramics [22], and biopolymers [23], with advances in nanomaterials such as nano-hydroxyapatite (nHAP), alumina-based nanocomposites, and poly-l-lactic acid (PLLA) composites [24,25,26,27]. Scaffold manufacturing techniques have also evolved, enabling large-scale production through solvent casting, particle leaching [28], freeze-drying, gas foaming, and electrospinning. Additionally, powder metallurgy and sol–gel techniques enhance the porosity and stability of these materials [28]. These innovations have strengthened tissue engineering in bone regenerative medicine by integrating precursor cells into biocompatible scaffolds combined with growth factors to stimulate cell proliferation, migration, and differentiation—critical processes in bone regeneration [29,30].

Scaffolds have been successfully incorporated into treatments that combine growth factors, autologous bone grafts, and stem cells to promote tissue regeneration and repair. Specifically, bone morphogenetic proteins (BMPs), platelet-derived growth factors, and vascular endothelial growth factors play a crucial role by stimulating stem cells and osteoblasts, thereby inducing regenerative processes at the defect site [31,32,33,34].

For example, autograft osteogenesis can be enhanced by doping with mesenchymal stem cells (MSCs) and biomaterials that mimic the chemical composition of bone, thereby improving the integration dynamics with bone tissue [35]. This approach is attractive because it overcomes the limited availability of these materials and increases their efficiency. Furthermore, they are easy to isolate and culture, which is appealing for the design of more advanced materials and strategies [36,37].

In this context, this article addresses the following question: How do biomaterials facilitate bone repair and regeneration, and what are their advantages over traditional methods? It achieves this through a comprehensive analysis of the most notable advances that have enabled the evolution of these materials and a meticulous study of synthesis techniques and chemical properties, facilitating a deeper understanding.

## 2. Literature Review

### Leading Causes of Bone Injuries: Trauma and Congenital Diseases

**Figure 1:**
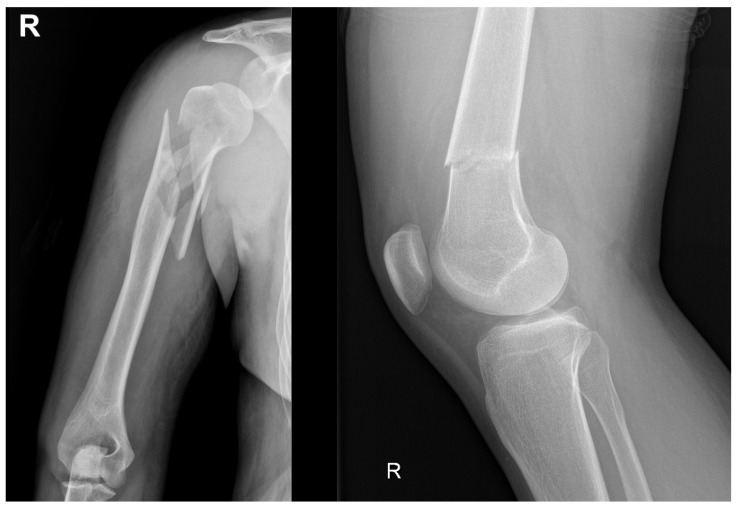
The (**left**) image shows a comminuted (multiple fragments) proximal humerus fracture. The (**right**) radiograph shows a transverse fracture of the distal femur. This is a patient from one of the authors (J.P.M.-C.): office at Fundación Valle del Lili, Cali, Colombia, with a signed consent form.

A fracture partially or completely disrupts a bone’s continuity [38]. The most common types of bone fractures include transverse, spiral, oblique, comminuted, and compression fractures, which are typically caused by either direct or indirect trauma. Less common, severe bone injuries have also been reported, such as stress fractures caused by excessive loading of the bone and more severe injuries of pathological origin that severely affect bone integrity. Figure 1 shows a comminuted fracture severely compromising the proximal humerus and a transverse fracture of the distal femur from a patient. Additionally, Table 1 summarizes the most common types of bone fractures, along with their corresponding descriptions and main characteristics; it also reports cases that require bone grafts and/or biomaterials to promote osteogenesis, depending on the severity of the fracture and the loss of bone tissue caused by trauma or disease.

**Figure 2:**
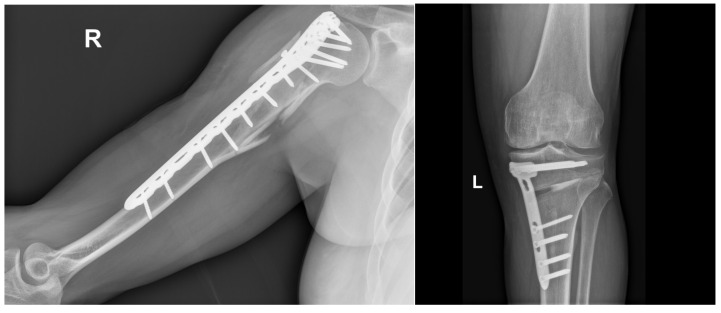
Comminuted humerus fracture that required open reduction, internal fixation with plate and screws, showing partial bone healing. This is a patient from one of the authors (J.P.M.-C.): office at Fundación Valle del Lili, Cali, Colombia, with a signed consent form.

Usually, orthopedic treatments based on inorganic materials are typically used to treat severe traumas like those in Figure 1 or, for example, the orthopedic treatment for reconstructing the proximal humerus (Figure 2). This reconstruction involves partial fixation and subsequent healing with tricalcium phosphate-based materials.

In addition, congenital causes have been reported to be associated with severe injuries. For example, genetic causes that favor the uncontrolled growth of immature osteocytes have been reported, leading to an accumulation that can cause malignant neoplasia and the development of osteosarcoma [38]. The pathogenesis of these conditions is influenced by various causes and/or genetic alterations, which contribute to 10% of reported cases, suggesting a genetic predisposition [43]. For example, multiple studies associated with these syndromes have suggested a hereditary origin; some of these syndromes have also been detected to have an autosomal dominant pattern of inheritance, in which one copy of the altered gene increases the risk of developing cancer [43]. Therefore, monitoring and early detection of immature osteocyte growth, which can lead to the development of neoplasia, is crucial.

On the other hand, certain syndromic disorders that increase the likelihood of tumor development, such as Rothmund–Thomson syndrome (RTS) and Werner syndrome (WS), are inherited in an autosomal recessive manner [44,45]. Similarly, the presence of non-hereditary somatic mosaicism has been documented in conditions such as polyostotic fibrous dysplasia and the McCune–Albright syndrome, as well as in enchondromatosis, which includes Ollier disease and Maffucci syndrome [46]. Likewise, there are numerous reports on various genetic syndromes causing tumors, which have specific characteristics that make their treatment difficult [47]. For example, the early detection of immature osteocytes in a particular area of bone tissue is challenging to identify and treat; in many cases, when this anomaly is detected, it is difficult to control, and it is necessary to resort to amputation of the affected limb, which generates physical limitations in patients [48,49,50].

**Figure 3:**
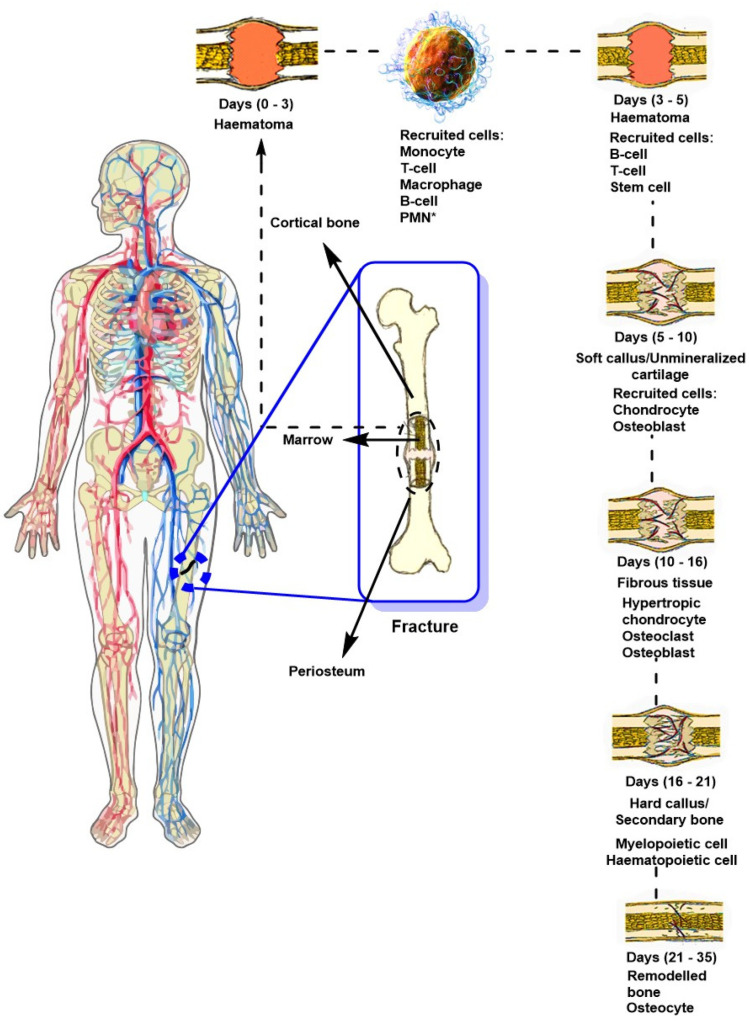
General diagram of bone healing and regeneration following a transverse fracture, highlighting the stages and cells involved in the healing process. PMN*: polymorphonuclear leukocytes [54,55].

Figure 3 shows the traditional process of bone regeneration after a transverse femoral trauma. The initial phase of this process involves a consolidation process that takes approximately three days, during which a hematoma forms. MSC cells play a crucial role, primarily due to their ability to produce growth factors and differentiate into osteoblasts, which promotes osteogenesis. In addition, the fracture microenvironment is conducive to promoting angiogenesis, which is attractive for applications in regenerative medicine due to its privileged immunological nature, facilitating the application of allogeneic therapies, thereby avoiding immunological rejection, promoting tissue regeneration, and expanding its potential for clinical application [51]. During the first few hours after a bone fracture, blood from the ruptured vessels enters the bony disruption, forming a clot known as a hematoma. The hematoma acts as a scaffold that enhances healing and ensures tissue repair occurs specifically at the injury site [52]. Subsequently, resident myeloid cells such as macrophages stimulate the influx of neutrophils, which are responsible for initiating the inflammatory response. At the same time, specific chemoattractants are secreted that promote the infiltration of immune cells such as macrophages, eosinophils, and mast cells, accumulating within the affected site [53]. Furthermore, some monocytes can differentiate into dendritic cells (DCs), which influence immune and adaptive responses and activate T cells. Subsequently, B cells emerge, secreting factors such as interleukin-10 (IL-10) and tumor necrosis factor alpha (TNF-α) that modulate osteoclast and osteoblast activity [52]. After this process, the formation of a soft fibrous tissue called a callus begins 5–10 days after the injury, which joins the ends of the injury. At this stage, the mechanical stability of the affected area is essential to ensure proper bone regeneration. This stage onsets 10 to 16 days after the bone fracture has occurred. Finally, the soft tissue production stage is fully completed in the maturation phase, and the affected area hardens. This happens 16–21 days after the fracture has occurred. Secondary bone is produced through the production of osteocytes, which occurs 21–35 days after the injury.

In this healing process, the periosteum (connective tissue or membrane on the surface of the bone) plays a crucial and dynamic role in bone consolidation by facilitating cellular activation. This tissue/membrane comprises two fundamental layers: an external fibrous layer and an internal cambium layer, which are essential in consolidating bone defects due to their high osteogenic cell content, promoting osteogenesis [56]. Between 3 and 5 days after the fracture (Figure 3), swelling and localized hemorrhage attract various immune cells and cytokines. The periosteum acts through the activation of mesenchymal stem cells and osteoprogenitor cells, initiating the repair of the vascular interruption through the activity of growth factors such as platelet-derived growth factors (PDGFs) and transforming growth factors beta (TGF-β) favored by the activity and differentiation of periosteal cells, giving rise to the bone callus and strengthening of the bone interruption [56]. In this context, unconventional methods that promote and improve the kinetics of bone healing in complex fractures are an attractive approach in regenerative medicine, as they can accelerate the healing process and strengthen the affected areas, avoiding morbidity in patients. For example, such is the case with implants with chemical compositions similar to those of bone, porous biomaterials that allow the controlled release of drugs and growth factors, among others.

## 3. Innovations and Applications of Biomaterials in Bone Repair and Regeneration

The efficient regeneration of bone defects, enabling the recovery of the affected limb while avoiding immune rejection and physical limitations, is a dynamic field in which various practical techniques have been reported for repairing problematic bone defects. Among the most significant traditional methods are bone grafts and transplants [57], which include autologous bone, such as iliac and/or fibular bone grafts, freeze-dried allografts, and xenografts [58]. Various fixation devices for these materials exist within the native host bone as supports, allowing space-filling and enhancing the diffusion of cells, growth factors, and minerals that promote regeneration [59].

Furthermore, various alternative therapeutic approaches include sophisticated implants used as internal prostheses in cases of bone resection caused by tumors [58]. Distraction osteogenesis can shorten a compromised limb, with or without the need for osteogenesis. In this case, the Ilizarov technique is recognized, which allows bone diffusion as an alternative to amputation of the affected limb [60].

**Figure 4:**
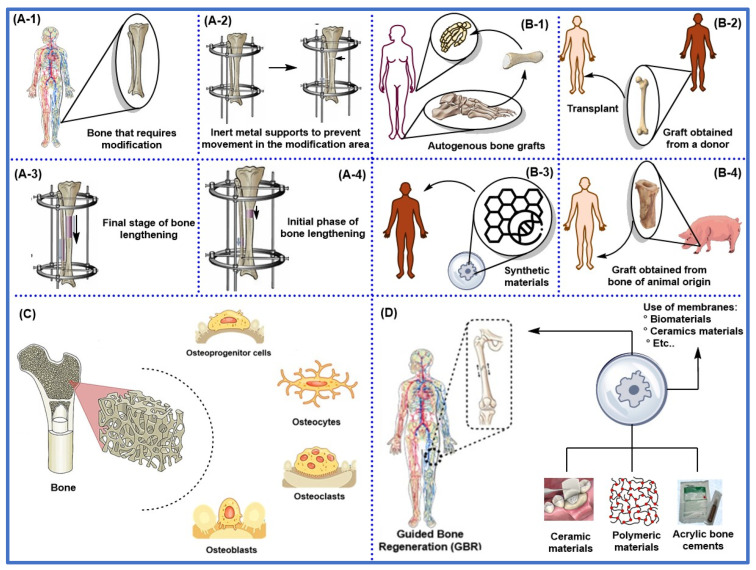
A general outline of conventional bone repair techniques, including (**A-1**–**A-4**) surgical methods, utilizes bone distraction to allow for gradual bone correction by separating two bone segments. (**B-1**) Autologous bone (bone fragments obtained from the same patient) is used in repairing and reconstructing lost or damaged bone tissue. (**B-2**) Allogeneic bone (obtained from donors of the same species) is used in bone transplants. (**B-3**) Organic or inorganic synthetic materials with chemical compositions similar to those of bone, which increase their compatibility and osteogenicity; (**B-4**) Xenogeneic bone (obtained from animals of different species), which increases the risk of rejection and infection. (**C**) Bone regeneration based on different types of bone cells to promote their growth or repair. (**D**) Bioactive materials, such as biomaterials or polymers, and bioinert materials, including ceramics and acrylic cement, are utilized to treat bone defects.

Figure 4 illustrates the most relevant conventional bone repair techniques, among which distraction osteogenesis stands out (Figure 4A), promoting new bone formation through the gradual and controlled separation of two bone segments. Other traditional approaches include first-, second-, third-, and fourth-generation methods (Figure 4B), encompassing autologous bone grafts (Figure 4(B-1)), allogeneic grafts (Figure 4(B-2)), xenografts (Figure 4(B-3)), and synthetic biomaterials with osteoconductive properties (Figure 4(B-4)). Cell-based therapies enhance bone regeneration (Figure 4C), and inert or bioinert materials improve healing and repair processes (Figure 4D).

In many cases, one of the main challenges in significant bone defects is that cells cannot migrate across the space created; instead, they require a solid foundation to generate new tissue and connect the fractured or damaged ends [61]. When fracture edges are separated or unstable, the healing process becomes ineffective, potentially leading to heterotopic ossification, also known as heterotopic pseudoarthrosis. Moreover, the body cannot regenerate independently in critical bone defects, making it essential to fill the gap to maintain limb alignment and length [5].

### 3.1. Nanostructured Materials: Manufacturing Methods and Clinical Applications in Bone Regeneration

Recently, the development of various nanostructured materials has been influenced by the advancement of nanotechnology, enhancing their effectiveness in bone regeneration due to their exceptional physical and biological properties that mimic the chemical composition of native bone, thereby favoring the integration of the material [62,63]. Chemically, these designed materials comprise ceramic nanoparticles such as hydroxyapatite and/or polymers that contribute to improving the mechanical properties and biological interactions with the chemical composition of bone [64]. The nanometric surface of these materials improves their osteoconductivity and osteoinductivity, facilitating bone growth orientation and stimulating stem cell differentiation toward an osteogenic phenotype—an essential factor for successful therapeutic outcomes in bone defects [65].

In a medical context, using various nanostructured scaffolds presents attractive advantages, including biodegradability and moderate biocompatibility that facilitate the growth of new bone. These materials are designed to ensure their biodegradation is non-toxic and coincides with the generation of new bone, thereby not impeding or obstructing its growth [66]. Moreover, the mechanical strength of these materials is suitable for conferring stability and withstanding loads during the consolidation of new bone tissue. Additionally, innovative methods have been reported to functionalize these materials, improving their biocompatibility and promoting the formation of new bone tissue. For example, doping with bioactive biomolecules such as peptides significantly enhances cell adhesion, favoring interactions with MSC cells and promoting their differentiation into bone cells, thereby improving bone consolidation [67]. Innovative techniques such as medical imaging, computed tomography (CT) scans, and magnetic resonance imaging (MRI) are essential for monitoring bone regeneration and ensuring proper bone healing.

The design of biomaterials with potential applications in tissue engineering requires sophisticated technologies, such as 3D printing and controlled deposition methods, to achieve the appropriate geometric distribution and pore size, thereby enhancing the precision and effectiveness of the materials [62,63]. However, one limitation to overcome is the sterilization of the materials, as these processes require complex methods, such as prolonged exposure to radiation and ethylene oxide, which could alter their biological and structural properties [68]. This highlights the importance of selecting suitable sterilization methods that preserve material properties while minimizing the risk of adverse reactions in the body. Therefore, carefully selecting fabrication and sterilization techniques is crucial to optimizing the performance of nanostructured scaffolds in bone regeneration, ensuring their safety and efficacy in clinical applications [69]. In this sense, sterilization techniques such as the use of radiation and the nature of biomaterials such as collagen or hydroxyapatite pose challenges for their application because the structures of the materials that are necessary for their optimal functioning are altered, such as porosity and functional groups, among others.

Table 2 summarizes relevant preclinical and clinical studies on the use of nanostructured scaffolds for bone regeneration. This table provides a detailed assessment of the materials used, modifications applied to the scaffolds, sterilization processes employed, and implantation sites in each study. Additionally, it discusses the observed bone regeneration outcomes, allowing for an analysis of the translational potential of each material and fabrication technique within a clinical context.

### 3.2. Conventional Techniques in Bone Regeneration and Orthopedic Implants: Bioinert, Bioactive, and Biodegradable

In orthopedic clinical practice, implant materials are classified into three generations based on their function. The first generation includes bioinert materials such as alumina (Al_2_O_3_), which has high mechanical strength and is commonly used to coat worn prosthetic surfaces and fill bone defects [6].

Second-generation materials include bioactive compounds, such as hydroxyapatite, known for its excellent osteointegration properties, as well as metal alloys like ASTM F136, a composition of 90% titanium, 6% aluminum, and 4% vanadium, chemically formulated as Ti6Al4V. These alloys are particularly valuable due to their Young’s modulus (longitudinal elastic modulus), which is similar to that of bone, promoting better integration. This generation also includes bioactive and biodegradable materials [87] that interact with the biological environment of the fracture, enhancing cellular responses and facilitating the integration between tissue and implant surfaces. Additionally, these materials are bioabsorbable, degrading in a controlled manner as bone regeneration progresses [88]. This group comprises metals, ceramics, and polymers designed to optimize osteointegration.

Finally, third-generation materials include bioglass and tricalcium phosphates (TCPs), which form a bone-like matrix and gradually degrade in the body during bone regeneration [89]. These materials are often combined with bone-growth-promoting compounds to enhance their effectiveness in dental implants, where various surface treatment techniques are required [90].

Biodegradable nanostructured scaffolds are designed to provide temporary structural support that promotes and stimulates the growth of bone cells. These properties are achieved through doping with bioactive ceramics such as hydroxyapatite and tricalcium phosphate, which have excellent biocompatibility and osteoconductive properties [91]. Additionally, hybrid matrices comprising various polysaccharides and metals, such as calcium, favor mechanical and biological properties [92].

These advancements aim to develop materials with mechanical properties like natural bone, strong enough to withstand surgical handling while integrating effectively with bone tissue. Furthermore, the degradation process of these materials must align with the healing time of the fracture or injury, as excessively fast or slow degradation could compromise the clinical effectiveness of the treatment [93].

### 3.3. Bone Grafts

Bone grafts are fundamental in regenerative medicine because they can replace and repair bone defects. There are several categories of bone grafts, each with its own properties and clinical applications. The main categories of bone grafts are described below.

#### 3.3.1. Autologous Bone

Autologous bone (grafts or fragments obtained from the same patient) is a key component in bone grafting due to its biocompatibility, osteoconductivity, osteoinductivity, and its content of living osteoblasts [94]. Its regenerative capacity makes it ideal for various clinical applications, particularly in dental implants, where rapid and effective bone formation is required [95]. It is also widely used in maxillary sinus lift procedures, complex bone defect reconstruction, and orthopedic treatments [96].

However, one of the main drawbacks of autologous bone grafting is the need for a second surgical site, which can increase postoperative morbidity [97]. Alternative options, such as allografts and synthetic biomaterials, are available to address this limitation, and they can be combined with autologous bone to optimize outcomes and reduce the adverse effects associated with bone harvesting.

#### 3.3.2. Allogenic Bone

Allogenic bone, also known as an allograft, is a bone graft obtained from donors of the same species, either from living individuals or deceased donors. Freeze-dried bone allograft (FDBA) is one of the most commonly used grafts in bone regeneration, particularly in dentistry and maxillofacial surgery [98]. Its production involves a freeze-drying process that reduces antigenicity, allowing for storage at room temperature. However, this process damages the osteoblasts in the tissue, limiting their osteoinductive capacity and prolonging the integration time with surrounding tissues compared to autologous grafts [99].

On the other hand, FDBA has shown more significant osteogenic potential due to the release of growth factors following demineralization. Nevertheless, its rapid resorption and the possibility of being encapsulated by connective tissue, leaving empty spaces in the bone cavity, pose challenges in maintaining the necessary volume for implants [100,101,102]. To overcome these limitations, combining allografts with bioactive factors, such as platelet-rich plasma (PRP), is emerging as a promising strategy to enhance the quality of newly formed bone and accelerate its integration with surrounding tissues [103].

#### 3.3.3. Xenogeneic Bone

Xenografts are based on non-living bone extracted from species other than the patient’s, typically obtained from bovine or porcine sources. However, there are serious challenges that must be addressed to ensure the success of the implantation. For example, there is a high probability of immune rejection due to antigenicity, and it may contain remnants of foreign organic matter in the recipient’s organism [57]. This situation can hinder the application of the material, triggering immune responses that prevent its integration. This material has been widely studied to overcome these limitations, as it is attractive for use as an implant due to its wide availability [57].

Various methods have been reported for processing this material to increase its sterility in this context. Among the most common methods is heat treatment, which eliminates any residue of organic matter present in the material, leaving only the inorganic part [104]. On the other hand, xenogeneic bone has attractive advantages compared to autologous and allogeneic bone. While autologous grafts require additional surgical procedures, xenogeneic grafts eliminate the need for these procedures, thereby reducing associated morbidity [105]. Additionally, their abundant availability makes them accessible in case large quantities are required.

Recently, the use of demineralized xenografts has been explored. These remove the mineral matrix while preserving bone morphogenetic proteins, thus maintaining more significant osteogenic potential [106,107,108]. These advancements enhance osteoinductive capacity and optimize graft integration with recipient tissues [105].

#### 3.3.4. Organic Synthetic Grafts

Synthetic bone grafts have gained prominence in regenerative medicine as viable alternatives to biological grafts. However, various synthetic–organic hybrid materials also present essential limitations that must be addressed to ensure implant success [109]. Polymeric grafts, such as those based on aliphatic polyesters, have been widely studied, with components like polylactic acid, polycaprolactone, and polyglycolic acid exhibiting favorable properties [110,111]. However, these materials release acidic substances during decomposition, as with polypropylene fumarate, which could negatively impact the integrity and microenvironment of bone tissue [112].

#### 3.3.5. Inorganic Synthetic Grafts

On the other hand, inorganic synthetic bone materials used as grafts include tricalcium phosphate (TCP), bioactive glass (BG), glass ionomer, alumina, calcium sulfate, and synthetic hydroxyapatite [113,114]. Bioactive glass stimulates bone regeneration, promoting osteointegration and bone formation.

Recently, new formulations have been developed combining different biomaterials to optimize their properties. For instance, combining bioactive glass with growth factors significantly enhances osteoinductivity and accelerates healing [115]. These materials aim to improve graft integration with the recipient bone and minimize complications associated with their use. Table 3 presents examples of the different types of bone grafts. While their benefits can be significantly enhanced through non-conventional regenerative medicine approaches, they also have certain limitations that, in some cases, could worsen the patient’s condition.

### 3.4. Demineralized Bone Matrix

The demineralized bone matrix (DBM) is a biomaterial derived from allogeneic bone, obtained through an acid extraction process that removes the mineral component, leaving a structure rich in type I collagen [119]. This process not only preserves the collagen matrix but also exposes osteoinductive factors, such as BMP proteins, enhancing its ability to stimulate bone formation compared to whole bone grafts [119]. Currently, DBM is available in various forms, including powder, granules, gels, putties, and pastes. However, its main limitations are its low mechanical strength and high porosity [120].

A study on craniofacial defect reconstruction revealed that DBM had the highest rate of residual defects compared to materials such as bone cement and autologous grafts [34]. This suggests it possesses valuable osteoinductive properties but is not an ideal scaffold for specific clinical applications. Composite scaffolds have been developed by combining DBM with materials such as polylactic acid (PLA) to enhance their effectiveness. These composites provide excellent structural stability and create an optimal environment for bone regeneration [121,122].

**Figure 5:**
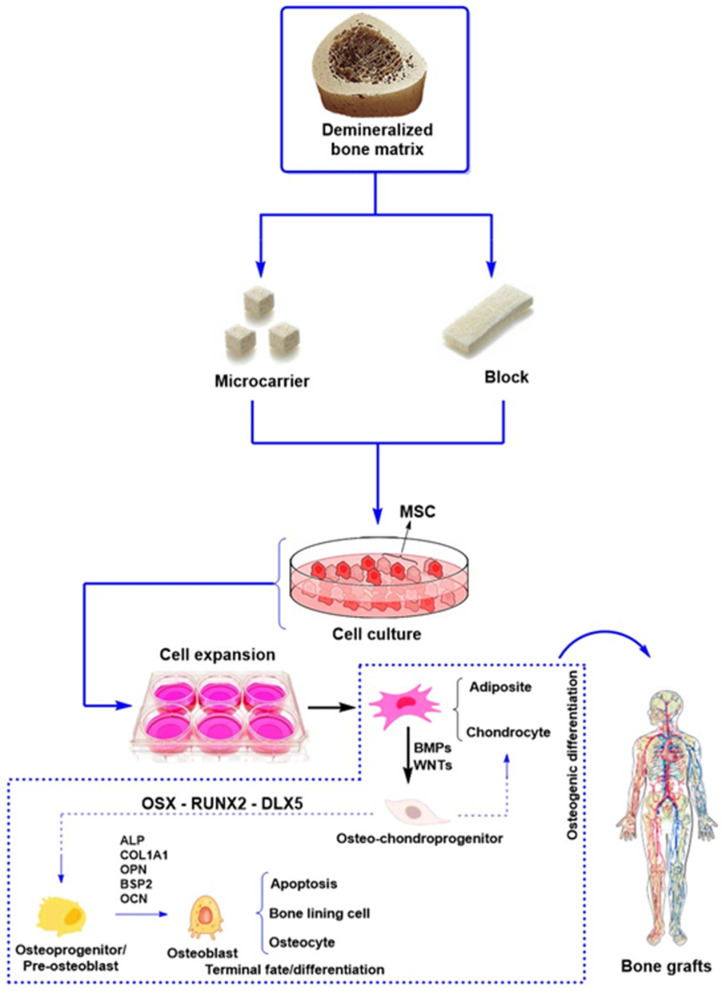
General scheme of the function of demineralized bone matrix to support stem cells and growth factors that promote bone regeneration [125].

Figure 5 illustrates the process of bone tissue demineralization for implants. DBM is available in two forms: microparticles and blocks. These structures are appropriate for MSC cell culture because they promote cell expansion, proliferation, and osteogenic differentiation [123]. Furthermore, Figure 5 highlights the traditional pathways of MSC differentiation into osteoblasts, emphasizing the pathways involving bone morphogenetic proteins and the Wnt/β-catenin signaling pathway. Key transcription factors such as RUNX2, osterix (OSX), and DLX5 regulate MSC differentiation into osteoblasts and chondrocytes.

In this context, osteoprogenitor cells transform into preosteoblasts, and the expression of osteogenic genes such as alkaline phosphatase (ALP) and COL1A1 is enhanced. These genes remain in the osteoblasts until they mature, along with markers such as osteopontin (OPN), bone sialoprotein II (BSP II), and osteocalcin (OCN). Finally, the osteoblasts undergo apoptosis and aggregate on the bone surface [124]. This process is essential for the regeneration and maintenance of bone tissue.

### 3.5. Ceramic Materials for Bone Scaffolds: Properties and Applications in Bone Regeneration

#### 3.5.1. Ceramics

Ceramics, defined as inorganic biomaterials designed to interact with biological tissues and support repairing, regenerating, or replacing damaged structures, are particularly valuable in bone and dental applications due to their biocompatibility and bioactivity. Materials such as calcium phosphate, hydroxyapatite (HA), and tricalcium phosphate (β-TCP) are widely used because of their safety and ease of sterilization, making them a viable alternative to autologous grafts [126,127,128]. However, these materials also present challenges. Calcium phosphate scaffolds have a very slow resorption rate, taking years to fully integrate with the surrounding bone, whereas tricalcium phosphate scaffolds can be resorbed within approximately 12 weeks [129,130].

Modifications have been made to the calcium/phosphate ratio and internal pore architecture to improve their osteogenic potential. For example, the use of monohydrated calcium phosphate (Ca(H_2_PO_4_)·H_2_O) with an ionic ratio (Ca/P) of 0.5 and a solubility of 18,000 mg/L and anhydrous monohydrated calcium phosphate (Ca(H_2_PO_4_)_2_) with a similar ionic ratio of 0.5 but with an improved solubility of 17,000 mg/L have been reported. Similarly, structural modifications such as dehydrated dicalcium phosphate (CaHPO_4_·2 H_2_O) have a higher ionic ratio of 1.0 and a solubility of 88 mg/L while anhydrous dicalcium phosphate has the same ionic ratio but a solubility of 48 mg/L; furthermore, octacalcium phosphate (Ca_8_(HPO_4_)_2_(PO_4_)_4_·5H_2_O) has a higher ionic ratio of 1.33 and a solubility of 8.1 mg/L [131]. These studies indicate that the higher the ionic ratio, the lower the material’s solubility, which impacts bone regeneration and degradation kinetics. That is, the increase in calcium in the material generates stable crystalline structures, which makes the dissolution or solubility of the material in the physiological environment difficult, affecting the degradation kinetics, unlike materials with low ionic ratio (<1), which have excess phosphates favoring the solubility of the material in physiological media and improving the degradation kinetics [131].

Scaffolds composed of hydroxyapatite and β-TCP have demonstrated a well-balanced combination of osteoconductivity and controlled resorption. Additionally, HA implants reinforced with collagen have improved stiffness and osteointegration in animal models, optimizing both mechanical properties and the environment for bone regeneration [132,133].

**Figure 6:**
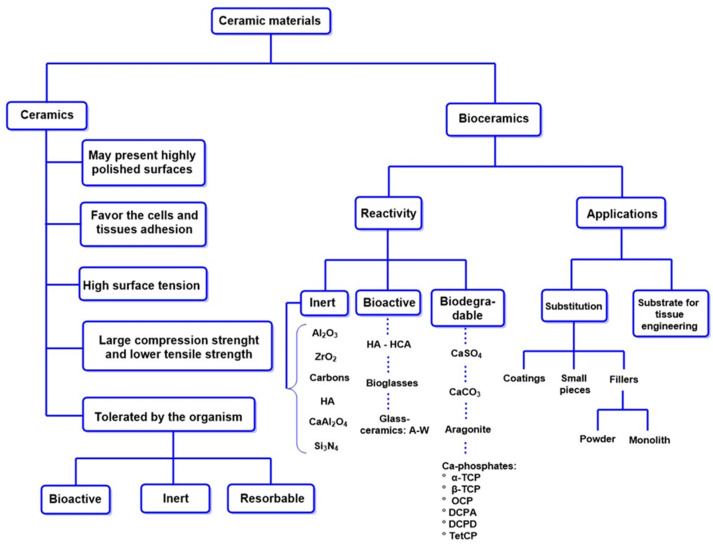
Properties and clinical applications of bioceramics. TCP: tricalcium phosphate. OCP: octacalcium phosphate. DCPA: anhydrous dicalcium phosphate. DCPD: dicalcium phosphate dihydrate. TetCP: tetracalcium phosphate monoxide. A-W: apatite-wollastonite.

A significant advancement in this field is the development of injectable hydrogels that combine collagen with calcium phosphate. These hydrogels enable the seeding of mesenchymal stem cells (MSCs) derived from umbilical cord tissue. These hydrogels promote osteoblastic differentiation and accelerate bone regeneration, representing an innovative strategy in regenerative medicine [134,135,136]. Figure 6 provides an overview of the main categories of ceramic materials [137]:

- Bioinert ceramics (Al_2_O_3_, ZrO_2_): These ceramics do not interact with bone tissue and belong to the first generation.
- Bioactive ceramics (calcium phosphates, bioactive glasses): Facilitate bone integration and possess osteoconductive properties, classified as second-generation materials.
- Third-generation ceramics: Combine features of both previous categories, offering improved mechanical and biological properties.
- Biodegradable ceramics: Incorporate biodegradable polymers to enhance performance in bone regeneration.

##### Inert Al_2_O_3_ and ZrO_2_ Ceramics in Bone Repair

Ceramic matrix composites (CMCs) have emerged as a promising alternative to traditional composites and monolithic ceramics in structural applications due to their exceptional properties, including excellent fracture toughness, chemical stability, and wear resistance [138,139]. Among the various CMCs, the ZrO_2_-Al_2_O_3_ composite has gained significant attention from researchers in this field due to its outstanding mechanical characteristics and the hardness of ZrO_2_. Furthermore, this material is highly resistant to mechanical stress, making it an attractive option for medical applications in areas that require high mechanical strength [140].

Furthermore, this compound exhibits exceptional mechanical properties due to its uniform distribution and ability to transform and integrate. Phase transformation prevents crack formation, increasing the effectiveness and efficiency of the material for treating bone defects [141]. However, some limitations must be addressed. For example, under stress conditions, this material can degrade, generating cracks in the structure and weakening it, which is particularly severe in orthopedic applications for bone reconstruction [142].

Despite its many advantages in medical applications, it has a significant disadvantage: its machining difficulty. However, recent advances have successfully enabled short-pulse lasers to optimize CMC processing, capitalizing on its thermal properties. These advances have successfully produced high-precision, high-quality materials used in orthopedic surgery [143,144]. These developments enhance graft integration with the host bone and minimize associated complications, thereby ensuring the long-term success and reliability of orthopedic implants [145].

##### Calcium Phosphates in Bone Repair: Bioactivity and Tissue Formation

Calcium phosphate (Ca(PO_4_)_2_) is a key component of hard tissues such as bones and teeth, and it has been extensively studied for its role in bone regeneration and clinical applications [126]. Biomaterials based on Ca(PO_4_)_2_ promote cell adhesion and proliferation by interacting with extracellular matrix proteins, thereby inducing the formation of new bone minerals. The bioactivity variability of these compounds depends on their chemical species, and their degradation properties influence the release of calcium and phosphate ions, altering the pH of the bone microenvironment and affecting the viability of osteoblasts and osteoclasts [146].

An important aspect is the high concentration of phosphate and calcium ions, which dynamically promote bone tissue mineralization and regulate the expression of genes involved in osteogenesis and the synthesis of essential proteins, such as type I collagen (Col-I), which confers stability and strength to the bone structure. Similarly, alkaline phosphatase (ALP) also plays a fundamental role in bone tissue regeneration through the hydrolysis of pyrophosphate. Among other proteins with similar functions are osteopontin (OPN), which plays an essential role in the mineralization of phosphates and their fixation to bone tissue; osteocalcin (OCN), which acts as a molecular marker of osteoblast aging; and transcription factors such as RunX2, which has a fundamental role in the regulation of osteoblast cell differentiation. Additionally, bone morphogenetic proteins play a crucial role in osteogenesis by promoting the differentiation of mesenchymal stem cells (MSCs) into osteoblasts [147,148]. The ability of these substances to modify the bone microenvironment makes them key components for treating bone defects with biomaterials that mimic the chemical composition of bone.

On the other hand, hydroxyapatite (HA: Ca_10_(PO_4_)_6_(OH)_2_) is the most abundant crystalline biomineral in bones, constituting approximately 70% of the dry weight of bone tissue. Its high stability and low solubility are surpassed only by fluorapatite (FAP) [149]. HA can form naturally or synthetically, a crucial factor in biomedical applications. While natural HA exhibits structural defects that incorporate vacancies and ions, synthetic HA can vary in structure and properties depending on the synthesis methods employed. Due to its stability, the hexagonal crystalline phase is preferred in biological environments [150]. The mechanical properties of HA improve according to the Ca/P ratio. For example, precipitated HA (Ca_10−x_(HPO_4_)_x_(PO_4_)_6−x_(OH)_2−x_ (0 < x < 1) has an ionic ratio between 1.5 and 1.67 and a solubility of 9.4, which presents a moderate crystallinity favoring the degradation kinetics during bone regeneration; that is, the physical process of disintegration and fragmentation is favored because of the biodegradation of the material through cellular processes. These characteristics are essential because the biodegradation processes of HA doped with an appropriate Ca/P ratio depend on a moderate crystallinity and solubility in the physiological environment [151]. Another outstanding example are the materials based on amorphous calcium phosphates (Ca_3_(PO_4_)_2_·nH_2_O (n = 3–4.5; 15–20% H_2_O)) which have an ionic ratio of 1.5 and a solubility between 25.6 and 32.8, which makes it ideal for the treatment of severe bone defects with loss of bone mass [131]. In addition, incorporating ions such as Mg^2+^, F^−^, Cl^−^, and CO_3_^2−^ can modify its behavior, enhancing its biological activity [152].

Similarly, tricalcium phosphate (TCP) is another widely studied CaP material due to its two crystalline phases: α-TCP and β-TCP [153]. While α-TCP is poorly soluble and does not precipitate in aqueous solutions, β-TCP is more soluble and can be synthesized through solid-state reactions, thermal conversion, and precipitation. Generally, β-TCP is obtained through heat treatment at 800 °C, which enables the precipitation of its crystalline form in solutions such as ethylene glycol and methanol, among others [154]. At temperatures above 1125 °C, β-TCP is converted into α-TCP, exhibiting improved properties. However, the versatility of β-TCP is more attractive in regenerative medicine due to its precipitation capacity in various low-cost and low-toxicity organic solvents, allowing for the production of α-TCP through heat treatment [155]. As demonstrated in recent studies, its ability to precipitate in organic solutions at low temperatures opens new possibilities for TCP synthesis under more controlled and biocompatible conditions [156].

A key aspect of bone regeneration is the recruitment of mesenchymal stem cells (MSCs) from the bone marrow to the site of the bone defect. Once there, these cells differentiate into osteoblasts, guided by the progressive degradation of Ca(PO_4_)_2_ materials, which release Ca^2+^ and PO_4_^3−^ ions [157]. The localized increase in ions above physiological levels promotes the differentiation of MSCs into osteoblasts, favoring the formation of new bone tissue [158]. That is, Ca^2+^ ions generate a cellular signal that regulates the regeneration of new tissue by inducing the formation of osteoblasts in the affected areas. Additionally, high concentrations of Ca^2+^ enhance the maturation and differentiation of MSCs, preosteoblasts, and osteoblasts, facilitating and promoting their migration to the affected areas. The increased intracellular Ca^2+^ ions promote membrane polarization, facilitating sustained cell migration [159]. This mechanism is essential because it encourages the formation of new cells through the generation of an appropriate microenvironment, as well as enhancing the efficiency of Ca(PO_4_)_2_ in bone integration. In this sense, these properties favor the integration of phosphates, making the material more efficient.

#### 3.5.2. Bioceramics

The classification of bioceramics is based on their reactivity rather than their chemical composition or crystallinity. Within the category of first-generation amorphous ceramics, there are glasses with the same chemical composition that, depending on their processing, can behave as bioinert, bioabsorbable, or bioactive materials [160]. Similarly, some ceramics with compositions identical to bioglasses may exhibit bioinert behavior, depending on whether they are produced through fusion or sol–gel methods [161]. A notable feature of bioceramics is their ability to form strong bonds with tissues, thereby promoting tissue integration [162].

However, their high mechanical strength, chemical stability, and bone regeneration capacity are limited, which limits their clinical applications [160]. On the other hand, second-generation bioceramics, such as calcium phosphates and bioglasses, are designed to enhance their interactions with bone tissues, facilitating osseointegration through the formation of chemical bonds with the tissue, which makes them attractive for use as coatings for implants and bone defects [163].

It is essential to note that third-generation bioceramics have been successfully utilized in the treatment of bone defects due to their porous structure, which facilitates the diffusion of bioactive molecules that stimulate the regeneration of the affected tissue. This represents a significant advance because it avoids amputation or replacement of the affected area, thus preventing physical limitations and morbidity in the patient. Similarly, the development and application of porous scaffolds play a crucial role in treating bone defects due to their ability to control and localize the release of drugs and bioactive molecules [137]. These multidisciplinary approaches have led to significant advances in the treatment of complex bone defects, enabling complete patient recovery and a breakthrough for public health [164].

In this context, Table 4 summarizes the most relevant ceramic biomaterials used in the treatment of bone defects. Among these materials, hydroxyapatite, bioglass, and tricalcium phosphates stand out, each with unique and attractive properties for use in regenerative medicine [165]. Large-scale production has been achieved through various synthesis approaches, including hydrothermal reactions, sol–gel synthesis, and innovative methods such as mechanosynthesis [166].

### 3.6. Bioglasses: Properties, Synthesis, and Applications in Bone Regeneration

Bioactive glasses represent a diverse class of materials used in bone regeneration, primarily in two osteogenic structures: porous glass–ceramics and glass–polymer composites, which are employed in scaffold systems [176]. Among these, calcium and sodium phosphate systems (SiO_2_-Na_2_O-CaO-P_2_O_5_) stand out [137]. One of the most significant materials is 45S5 bioactive glass, an FDA-approved biomaterial widely utilized in tissue engineering and bone repair. Its versatility lies in modifying its composition by incorporating therapeutic ions such as fluoride, magnesium, boron, strontium, zinc, silver, and cobalt. These modifications enhance its integration with biological tissues and enable specific clinical adaptations. Its applications range from implantable particles to scaffolds for bone regeneration, making it a key material in advanced biomaterials [137].

**Figure 7:**
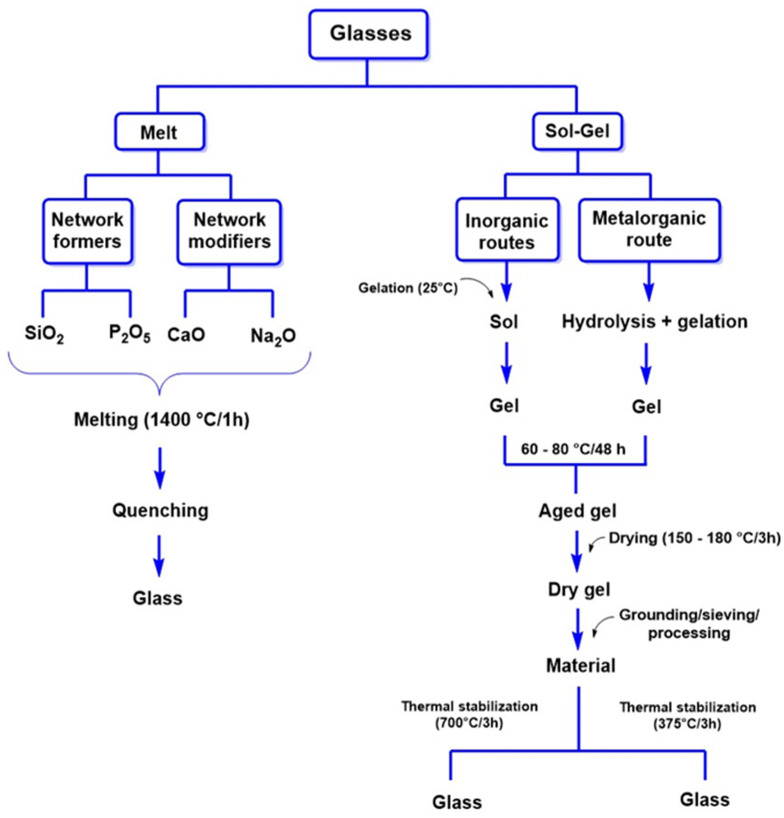
Key methods for synthesizing bioglasses for regenerative medicine applications. Adapted from Vallet-Regí et al. [161].

Regarding its synthesis, bioactive glasses can be produced through conventional methods, which involve cooling molten minerals where SiO_2_ and P_2_O_5_ act as network formers, while CaO and Na_2_O function as network modifiers, as illustrated in Figure 7. However, the sol–gel method has gained prominence recently, enabling the production of bioactive glasses with high purity, homogeneity, and improved textural properties. Additionally, this method operates at lower temperatures than traditional melting techniques, enhancing process efficiency.

These compositions have shown great promise in tissue engineering, as they mimic the chemistry and architecture of the bone extracellular matrix. In particular, the silicon in these glasses plays a key role in regulating angiogenesis and gene expression associated with osteogenesis while stimulating the production of growth factors by osteoblasts [177]. Recent studies have confirmed that silicate-based structures can enhance bone formation by incorporating them into bioceramics, thereby increasing bioactivity and osteoinductive effects [178,179].

For instance, combining silicon with hydroxyapatite (HA) has been shown to demonstrate superior bone formation compared to using HA alone. However, these hybrids exhibit low mechanical strength, limiting their application in load-bearing areas [180]. An up-and-coming alternative is calcium silicate, which promotes the osteogenic differentiation of various cell lines, including bone marrow-derived mesenchymal stem cells (BMSCs), and possesses proangiogenic properties. A notable feature of these materials is their ability to alter the bone microenvironment and induce defect regeneration without the need for exogenous bioactive molecules, which is attractive for enhancing traditional orthopedic therapies [181].

Research has recently focused on silicate bioactive glasses and ceramic scaffolds with dual capacity to induce osteogenesis and angiogenesis [182]. This property is particularly relevant as it could simplify treatments by eliminating the need for multiple biomaterials or external factors. Moreover, advances in the fabrication of mesoporous glasses have enabled the development of structures with macropores and highly organized nanopores, improving their bioactivity and facilitating the incorporation of therapeutic ions and biomolecules [183]. These materials accelerate bone formation and optimize the integration with the surrounding tissue.

#### Nanobioglasses: Properties and Applications in Regenerative Medicine

Nanobioactive glasses are materials with particle sizes smaller than 100 nm whose unique physicochemical properties enable a rapid bioactive response and superior cellular interaction compared to traditional bioactive glasses. Their high surface area, fast biodegradability, spherical morphology, and enhanced release of bioactive ions have established them as one of the most promising alternatives in regenerative medicine thanks to their exceptional bioactive and mechanical properties [184]. In this sense, nanobioactive materials play a fundamental role in the regeneration of bone defects, as they stimulate the formation of hydroxyapatite on the surface of the affected areas. This represents a significant advance in improved integration into bone tissue, opening the door to multiple innovative applications, such as advanced orthopedic implants for bone tissue regeneration in maxillofacial treatments [185]. Furthermore, by releasing essential ions such as calcium (Ca^2+^) and phosphate (PO_4_^3−^), these materials stimulate osteogenic activity, contributing to improved bone integration and accelerating the recovery process.

Nanobioactive glasses can be synthesized through various methods, with sol–gel and melt-quenching being the most widely used due to their ease of fabrication and ability to generate highly porous structures that promote cellular infiltration and vascularization of the regenerated tissue [184]. Regarding their production, nanobioactive glasses can be manufactured using different methods, particularly the sol–gel and melt-quenching approaches, which are valued for their simplicity and their ability to create porous structures ideal for cell infiltration and blood vessel formation in regenerated tissues [186]. On the other hand, recent techniques, such as spray pyrolysis and microemulsion synthesis, have enabled the improvement of both the mechanical resistance and biocompatibility of these materials [184].

Combining nanobioactive glasses with natural polymers such as chitosan and cellulose has driven the development of functional biomaterials for hard and soft tissue regeneration [187,188]. These combinations have enabled the design of scaffolds, films, coatings, and dressings, enhancing the material’s bioactivity while optimizing its mechanical properties for better integration with the host tissue [189]. Composite scaffolds facilitate cellular migration and vascularization, key elements for the effective recovery of injuries [190].

Table 5 presents the most used polymers in combination with nanobioactive glasses for bone regeneration. Among them, alginate stands out for its ability to provide controlled drug release, enabling targeted therapies in specific areas. Functionalized alginate microspheres incorporating collagen and hyaluronic acid have been developed to release growth factors, such as IGF-1 and TGF-β1, which are essential for bone tissue proliferation and maintenance [191]. Furthermore, poly(hydroxybutyrate-co-hydroxyvalerate) (PHBV) has shown excellent performance in three-dimensional matrices for chondrocyte growth. At the same time, chitosan facilitates the formation of bioactive nanofibers, thereby promoting nanobiomineralization and stimulating the development of the extracellular matrix. Likewise, the combination of chitosan (CS), gelatin (G), nanobioactive glasses (NBGs), and reduced graphene oxide (GO) has demonstrated high cytocompatibility with MG-63 cells, confirming its relevance in regenerative medicine [192].

### 3.7. Synthetic and Natural Polymers Used in Bone Tissue Regeneration

Polymer-based materials are highly valued in various research fields due to their design flexibility, biodegradability, and biocompatibility, among other properties. These properties make them widely used as scaffolding materials in bone tissue engineering [196]. Natural polymers such as hyaluronic acid [202], xanthan gum [203], alginate, and lignin are frequently employed [204]. However, there are significant limitations to the use of natural polymers, such as low mechanical strength and poor thermal properties.

There are reports of synthetic polymeric materials that have yielded promising results. For example, poly(lactic-co-glycolic acid) (PLGA) [205], polycaprolactone (PCL) [206], and polyethylene glycol (PEG) [207] have been successfully applied in the treatment of bone defects, presenting excellent mechanical properties. However, they have severe limitations, such as hydrophobic surfaces that can interrupt osseointegration, a fundamental aspect that must be addressed to improve their effectiveness [208]. One synthetic polymer that does not present these drawbacks is polylactic acid (PLA), a biomaterial approved by the U.S. Food and Drug Administration (FDA), which provides controlled biodegradation, high biocompatibility, and adequate mechanical strength. Its versatility has enabled clinical applications in absorbable sutures, drug delivery scaffolds, and resorbable bone fixation devices, promoting fracture healing. However, its limited osteoinductive properties have restricted its use in craniofacial bone regeneration [209].

To overcome these limitations, combinations of natural and synthetic polymers with ceramics have been explored to develop materials with tailored properties for tissue engineering applications [210]. Additionally, innovative strategies have been implemented to enhance the osteoinductive properties of polymeric scaffolds. For example, combining PLA with other biomaterials such as PLGA has improved its ability to induce bone formation [211]. Furthermore, the use of porous scaffolds has demonstrated improvements in vascularization and cellular migration, which are essential for successful bone regeneration [212].

Another polymer of great interest is poly(propylene fumarate) (PPF), an unsaturated linear polyester known for its biodegradability, biocompatibility, and osteoconductivity. These properties make it a promising material for craniofacial bone regeneration [213]. Its injectability and suitable mechanical strength make it particularly well suited for such applications. It is essential to note that a monomer accelerator, such as N-vinylpyrrolidone, is often necessary to optimize its performance and facilitate the crosslinking and curing of the fabric [214].

Recent studies have reported biphasic composites, such as PPF, which successfully incorporate cross-linked microparticles [210]. This approach optimizes the material’s strength and increases its surface area, thereby enhancing injectability and effectiveness compared to other materials, including traditional bone cement doped with polymethylmethacrylate (PMMA), which is commonly used in clinical procedures. In this sense, the application of PPF has represented a significant advance in bone tissue regeneration [215].

On the other hand, PPF has been utilized for the development of copolymers using PCL (a polyester characterized by its flexibility and adjustable biodegradation rate, which is dependent on its molecular weight) for the synthesis of scaffolds that promote osteoblast maturation and MSC cell differentiation [216].

These biomaterials exhibit enhanced cellular adhesion and offer functional support, surpassing that of synthetic polymers. However, their use faces challenges, including limited control over mechanical properties, potential immunogenicity, and restricted availability, which can increase production costs and limit accessibility [217].

#### 3.7.1. Synthetic and Natural Polymers as a Basis for Nanobiocomposites in Bone Regeneration: Functionality and Advanced Applications

**Figure 8:**
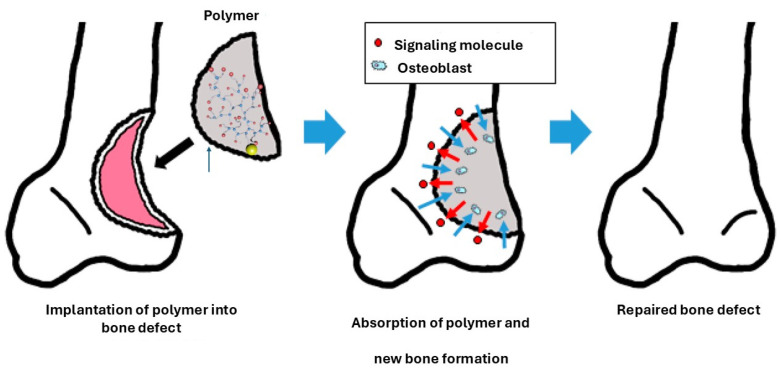
Using biodegradable polymers as fillers in bone defects promotes regeneration by releasing signaling molecules, such as osteoblasts. Reproduced with permission from (Aoki and Saito, *Pharmaceutics*, published by MDPI, 2020) [218].

Both synthetic and natural polymers play a crucial role in the structural framework of bioactive scaffolds used in bone regeneration [218]. When filling a bone defect, the signaling molecules embedded in the scaffold are gradually released to stimulate the formation of new bone tissue. Cells such as osteoblasts migrate toward the scaffold, initiating bone tissue formation and degrading the biodegradable scaffold. Over time, the scaffold is wholly replaced by autologous bone, ensuring effective and natural integration with the surrounding tissue, as illustrated in Figure 8.

Biopolymers, such as dextran, chitosan, and collagen, facilitate the incorporation of nanoparticles, thereby enhancing the biocompatibility and functionality of hybrid materials. For instance, dextran, a natural polysaccharide with a branched glucose molecule structure, is highly valued for its low immunogenicity and chemical stability. Its ability to form hydrogels makes it an ideal material for sustained drug delivery systems, optimizing treatment efficacy and minimizing side effects. When combined with bioactive nanoparticles, dextran creates a bioactive environment that promotes the proliferation and differentiation of stem cells into osteoblasts, thereby laying the foundation for the development of functional nanocomposites [219].

Successful integration of diverse biopolymers and nanocomposites requires a thorough evaluation of their ability to mimic the natural bone matrix. This aspect is key to achieving an adequate balance between stiffness and elasticity. Materials such as chitosan and collagen have proven effective in forming porous matrices, which is essential for vascularization and the transport of nutrients and key elements in the development of more specialized hybrid nanocomposites [220]. Furthermore, research on modern bioactive scaffolds focuses on chemically modifying these polymers to improve both their biocompatibility and functionality [221].

It is essential that polymers used in bone tissue regeneration have degradation rates comparable to those of new tissue regeneration and that the products generated by biodegradation are nontoxic and do not alter the microenvironment, thereby reducing immune responses and ensuring integration with the new bone tissue [222,223]. Fortunately, several investigations have focused on improving these characteristics by incorporating various bioactive compounds that stimulate osteogenesis [224,225]. Research targeting these structural modifications plays a fundamental role in the efficacy of these materials in regenerative medicine, ensuring the complete healing of various bone defects [226]. For additional inflrmation, refer to [227,228,229].

#### 3.7.2. Advances in Potential Nanobiocomposites for Regenerative Medicine

Nanofillers and biomolecules doped with nanobiocomposites represent an innovative approach to treating bone defects, offering significant advantages over traditional methods. For example, polymeric nanobiocomposites obtained from natural sources, such as chitosan, have not only demonstrated exceptional biocompatible properties but are also abundant and easily accessible. CS, obtained from chitin extracted from the exoskeletons of crustaceans, consists of β-1,4-linked N-acetyl-D-glucosamine and D-glucosamine units, making it structurally similar to the glycosaminoglycans (GAGs) found in bone. This similarity enhances bone regeneration, while incorporating nanohydroxyapatite (nHA) into CS composites significantly improves their mechanical and biological properties, providing a robust and bioactive scaffold for bone growth [230,231,232].

The CS/nHA structure, obtained through sol–gel processes and the self-assembly of nHA during film drying, has demonstrated that a higher nHA content increases surface roughness and hardness, thereby improving the efficiency of bone regeneration. However, excessive nHA content may accelerate CS thermal degradation up to 300 °C due to more efficient heat transfer, but the degradation rate decreases beyond this temperature. Balancing nHA content and thermal stability is crucial to optimizing the properties of the nanobiocomposite [233,234].

Furthermore, the functionalization of nanofillers has been reported to improve the properties and characteristics of polymer-based scaffolds significantly. For example, the functionalization of carbon nanotubes with Col-I and hydroxyapatite-based scaffolds has been reported. These functionalized materials serve as nucleation sites for bone, promoting tissue mineralization [235]. Titanium dioxide (TiO_2_) has also been incorporated into polymeric scaffolds based on silk fibroin (SF), thereby enhancing their mechanical properties and promoting cell interactions. Halogenated TiO_2_ nanoparticles (nTiO_2_-F) have demonstrated biocompatibility and enhanced scaffold bioactivity, although excessive concentrations can negatively impact mechanical properties due to uneven dispersion within the polymer matrix [236].

The combination of cellulose nanocrystals (CNCs) and halloysite nanotubes (HNTs) in xanthan gum (XG) and sodium alginate (SA) scaffolds has achieved uniform nanofiller dispersion, generating an interconnected porous morphology with high porosity [237,238,239]. These characteristics have improved the composite’s storage modulus and mechanical strength, enhancing cellular compatibility and adhesion, which are crucial for effective bone repair.

Furthermore, the development of fibrous scaffolds composed of PLGA/HA, synthesized via melt electrospinning, has enabled the incorporation of BMP-2, a key growth factor in bone regeneration, using polydopamine (PDA) as a polymeric bridge [240]. PDA coating on the PLGA/HA scaffold surface increases roughness, facilitating cell attachment and enhancing hydrophilicity, promoting excellent cell adhesion and proliferation. PDA’s flexibility allows for the integration of physical and chemical signals on the scaffold surface, thereby optimizing its bioactivity. Additionally, BMP-2 immobilization via PDA has been shown to provide sustained growth factor release, thereby enhancing MC3T3-E1 cell proliferation and promoting bone mineral deposition, which confirms the effectiveness of these nanobiocomposites for bone repair applications [241].

The growing development of TiO_2_ and poly(ester urethane) urea (PEUU)-doped scaffolds has been an innovative strategy that has allowed the expansion of the application of this material, which is obtained by electrospinning and has facilitated the production of fibers with diameters less than 1 μm, which is interesting to improve the mechanical resistance and its structure [242]. PEUU can also enhance the interfacial bonding with physiological matrices through functionalization with nTiO_2_; this functionalization improves the mechanical resistance of the material and the elastic modulus of the material in a ratio of (1:1), with an increase of 53% in Young’s modulus, which is significant evidence of the improved load capacity and structural stability [242]. The uniform distribution of nanoparticles in the polymeric material is crucial for enhancing the mechanical resistance and biocompatibility of the scaffold, which is essential for its applications in orthopedic treatments [243,244].

The combination of ceramic materials and polymers enables the development of hybrid scaffolds that optimize both mechanical and biological properties, providing innovative solutions in regenerative medicine. However, it is essential to consider the limitations and challenges associated with these methods. For example, the production and application of nanomaterials can be costly and technically complex, limiting their accessibility [245]. Additionally, the biocompatibility and potential long-term toxicity risks of specific nanomaterials must be carefully evaluated before their clinical application. Despite these challenges, the advantages—such as personalized treatments and the ability to overcome the limitations of traditional grafts—make these methods highly promising for future clinical applications in regenerative medicine [246].

In this sense, the degradation patterns of various biomaterials are crucial to their success in orthopedic medicine. This implies personalizing and/or adapting the biomaterial to the fracture type. For example, complex fractures or those with high mechanical load require biomaterials with good mechanical stability, such as fluoroapatite (Ca_10_(PO_4_)_6_F_2_) or tetracalcium phosphate (Ca_2_(PO_4_)_2_O), which have an ionic ratio of 1.67 and 2.0, respectively, and solubilities of 0.2 and 0.7. Due to the high mechanical strength of these biomaterials, the dissolution kinetics in the physiological environment is slow and the release of Ca^2+^ ions is slower, controlled and localized, which favors the activity of osteoblasts [247]. In the case of larger bone fractures and loss of bone tissue, using biomaterials with slower biodegradation kinetics is preferable to support the affected tissue and the new tissue in formation during longer recovery periods. Furthermore, doping with growth factors is preferable; biopolymers and bioceramics are essential to meet these requirements [247].

##### Controlled-Release Systems of Bioactive Molecules for Bone Regeneration

A fundamental aspect of biomaterials and bioactive materials is their multifunctional properties, such as their ability to induce osteogenesis and angiogenesis and their positive antibacterial and carcinogenic properties [248]. This multifunctionality can be induced in various materials through various approaches. For example, doping various materials with therapeutic ions is common in orthopedic procedures; however, these ions are insufficient for complex trauma [249]. For this reason, future research should focus on improving the multifunctional properties of these materials.

Several authors have reported significant progress in developing innovative materials such as nanobioglasses. These materials have a good surface area and larger pore volume than traditional bioglasses, making them attractive for the localized release of potential molecules for bone-generating therapies [250]. For example, tetracyclines are attractive molecules for bone regeneration therapies, such as doxycycline (DOX, a semi-synthetic derivative of oxytetracycline), which has been shown to stimulate osteogenesis and osteoblast apoptosis, regulate inflammatory bone resorption, and osteoclastogenesis [251,252].

In this sense, the load of bioactive molecules in the materials is a fundamental aspect that must be considered; that is, a better release of bioactive molecules and growth factors is obtained in materials with specific structural properties and characteristics [253]. For example, the following aspects are fundamental: (1) the materials or release systems must be biocompatible or bioinert, (2) the interaction between the materials and the molecules must not be strong in the sense of not generating new bonds that prevent their release, which facilitates their diffusion in the material, (3) it must be degradable in the microenvironment and the degradation products must not be toxic, which conditions the release kinetics of the molecules, (4) in some cases, it is desirable that the materials present a certain degree of swelling, which facilitates the release of the molecules [254,255].

In this context, cellulose-derived biopolymers such as hydroxyethylcellulose have been used for the controlled release of DOX in autogenous bone to reconstruct severe bone defects, as reported by Lucateli et. al. The authors used a gel based on the biopolymer natrosol (a hydroxyethylcellulose) at 10% for the controlled release of DOX in autogenous bone for the reconstruction of critical bone defects, demonstrating 38.59% bone formation after 8 weeks [256]. Additionally, simvastatin administration has been shown to enhance osteoblast activity, improving bone healing in treating femoral fractures. Furthermore, its topical application has been investigated in animal models for induced fractures, demonstrating promising results [257].

**Figure 9:**
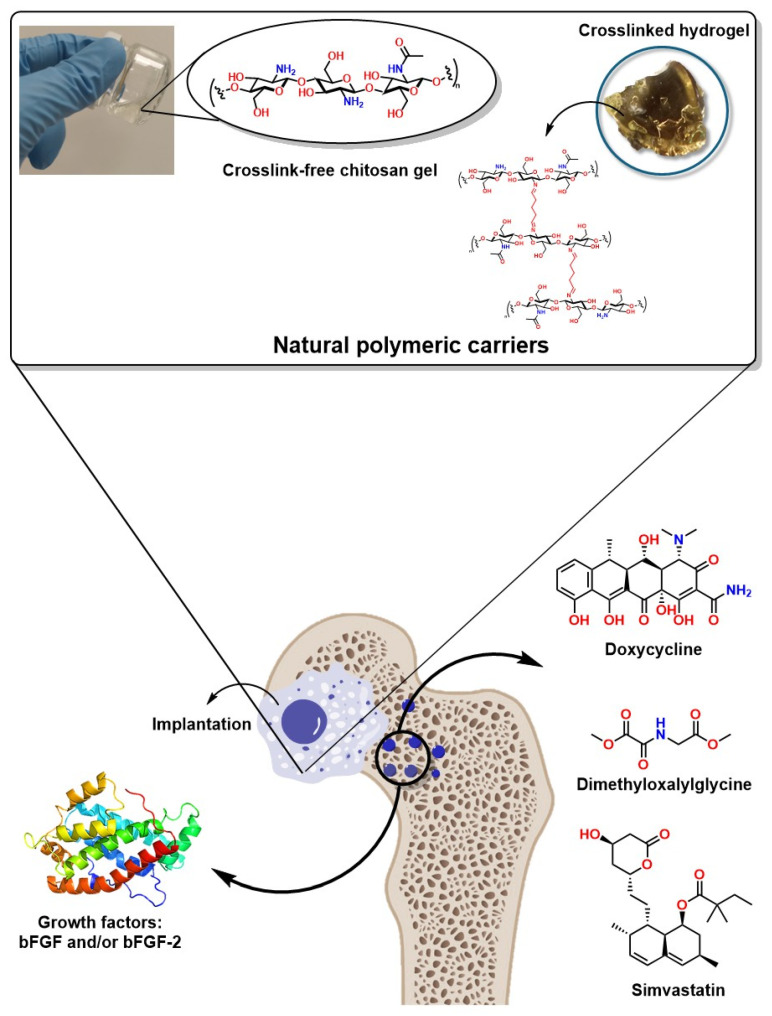
Schematic representation of drug and growth factor delivery systems through polymeric gels and cross-linked scaffolds (hydrogels).

Furthermore, mesoporous bioactive glass scaffolds have also been reported to enhance the release kinetics of drugs such as dimethyloxalylglycine (DMOG) and dexamethasone (DEX), significantly improving osteogenesis through the stimulation of alkaline phosphatase activity and osteoblast gene expression [253]. Moreover, DMOG (a low-molecular-weight molecule) with inhibitory activity and readily cell permeable by HIF-PH through the hydroxylation of specific proline residues has been successfully implemented in mesoporous systems such as bioglass; this mechanism stimulates HIF binding to von Hippel–Lindau tumor suppressors, promoting their degradation, generating a hypoxic microenvironment and acting as a proangiogenic compound [253]. Furthermore, growth factors such as bFGF and/or bFGF-2 in gelatin/β-TCP gels with porous poly(L-lactide-co-ε-carpolactone) (PLGC)/β-TCP membranes have been shown to increase bone volume, especially in fractures with bone loss [258]. Growth factors such as VEGF and TGF-β have been shown to promote bone growth in maxillofacial surgeries in bone filler impregnation applications, favoring neovascularization [259]. Figure 9 shows an idealized system for the controlled release of drugs and growth factors in a controlled manner in bone defects to promote their regeneration.

These advances represent an excellent approach not only as more efficient systems for bone regeneration and the controlled and localized release of bioactive molecules, but also as they increase the probability of success and decrease the risk of infection at surgical sites. For example, doping and/or coating bone implants with Doxycycline has prevented infections in vulnerable patients or those with weakened immune systems. Similarly, doping or coating bone implants with Metformin has facilitated the recovery and osseointegration of implants in patients with type 2 diabetes mellitus (DM2) and has presented excellent results of osteogenic differentiation in mesenchymal stem cells through the AMPK/BMP/Smad signaling pathway [260]. Hydroxyapatite doping with simvastatin in bone coatings and fillers has also demonstrated high effectiveness, preventing biofilm formation and spreading diseases associated with bone implant infections [261].

##### Injectable Cell Therapy for Bone Fractures

Several preclinical studies have demonstrated that transplantation or intravenous administration of bone marrow mesenchymal cells is potentially beneficial for correcting various bone marrow disorders. For example, Horwitz et al. studied the clinical response to bone marrow transplantation in children with severe osteogenesis imperfecta (a genetic disorder characterized by severe bone deformities, bone fragility, growth retardation, osteopenia, and the presence of defective type I collagen). The study revealed that this method significantly improved bone mineralization, linear growth, and mechanical resistance to bone fractures in children after six months of age [262]. Furthermore, the treatment of severe bone defects caused by trauma or diseases that affect bone integrity through these methods of injectable cell administration at the affected site has demonstrated significant benefits in severely affected patients. For example, it has been effective and has improved recovery times in patients with nonunion who required percutaneous autologous bone grafting [263].

In recent decades, traditional bone repair or healing therapies have demonstrated a highly multidisciplinary approach, utilizing a wide variety of natural and synthetic materials that have successfully promoted and enhanced osteogenic and angiogenic responses. For example, 45S5, 58S, and S53P4 bioglasses have been especially attractive in orthopedic therapies [264]. Furthermore, collagen-enriched bioactive glass scaffolds improve the mineralization of affected bone tissue, enhance the cell proliferation of MC3T3-E1 cultured on their structure, and increase alkaline phosphatase (ALP) metabolic activity [265].

A crucial characteristic of these materials is their chemical structure. Their structure must be similar to or compatible with physiological tissues to increase their biocompatibility and promote cell growth, improving their viability and retention [266]. Several studies have reported innovative methods for developing these materials, among the most notable being the decellularization of biological matrices and/or tissue immunomodulation [267]. Techniques such as electrospinning are attractive because they mimic the structure of the extracellular matrix, favoring its integration, or triphasic culture techniques that mimic physiological orthopedic interfaces. However, these techniques can be optimized through microtopography to favor signals for cell differentiation [268,269].

Poly(dimethylsiloxane) (PDMS), a silicone-based bioinert, is a suitable microenvironment for cell attachment, allowing cell proliferation. However, it does not promote cell differentiation, so further studies are needed to focus on these advances [270]. In this sense, the structural characteristics of the various polymers and materials used for bone regeneration influence cell growth and differentiation through growth factors, bioactive molecules and/or ions, and structural rigidity [271]. An attractive approach is using these materials for the controlled release of growth factors such as PDGF, BMPs, and VEGF, which activate mesenchymal stem cells, accelerating bone tissue recovery through the production of osteoprogenitor cells [272].

This therapeutic approach has improved the healing process of at least 60 successfully reconstructed femurs, avoiding morbidities in patients, according to the report of Kitoh et al. [273]. This approach has also successfully treated bone tumors (osteosarcomas) through cell growth in bone fillers such as hydroxyapatites and coatings such as chitosan scaffolds. This approach significantly improved the affected area’s recovery time and bone tissue recovery [274]. Furthermore, this approach has improved the recovery of patients with early vascular necrosis of the femoral head, avoiding amputations and morbidity. Moreover, this approach has dramatically enhanced the incorporation of heterologous bone into complex bone implants [275,276].

## 4. Smart Stimulus-Responsive Biomaterials for Bone Regeneration

In regenerative medicine, innovative stimulus-responsive biomaterials have emerged as a significant innovation in bone regeneration. These materials are designed to respond to specific physiological conditions, enhance osteoconduction and osteoinduction, and facilitate drug-controlled release and growth factors. A notable example is the development of hybrid biodegradable and bioinert scaffolds, which interact with mesenchymal stem cells (MSCs) and stimulate osteogenic processes by releasing bioactive molecules in response to changes in temperature, pH, or electrical signals [277].

**Figure 10:**
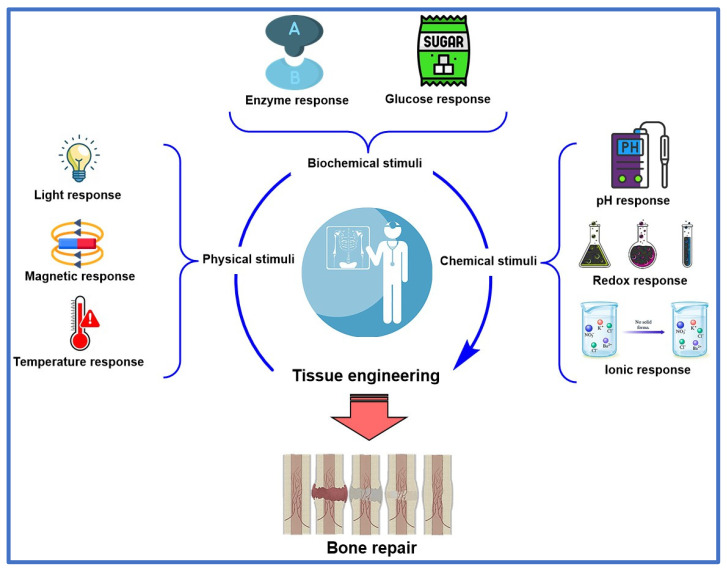
Some types of materials that respond to external stimuli promote bone regeneration.

The structure and chemical composition of these materials are essential to ensuring their effectiveness. For example, hydrogels used in regenerative medicine must mimic the extracellular matrix of bone tissue to promote osteogenic differentiation of MSC cells and osteocompatibility. Furthermore, these structures must be graded over time, similar to tissue regeneration, to prevent material accumulation and interference with the healing process. However, traditional hydrogels in this application have prolonged degradation times, compromising successful tissue healing and minimizing the risk of immune rejection. Figure 10 illustrates the most relevant types of materials utilized in regenerative medicine that have the potential to respond to external stimuli, thereby promoting bone regeneration.

In contrast, stimulus-responsive hydrogels have overcome these limitations by reacting to physiological variations, such as changes in temperature or pH, accelerating their degradation and the release of bioactive compounds. For instance, a thermoresponsive hydrogel can release growth factors at the optimal moment, enhancing the effectiveness of bone regeneration treatments [278].

Surface modification of biomaterials is a relevant aspect of regenerative medicine. These modifications are designed to achieve superior adhesion of biomaterials to tissue and promote cell proliferation. Doping or coating biomaterials with polydopamine (PDA) is a highly relevant approach in this field, as it enhances surface roughness and hydrophilicity, favoring cell adhesion. Furthermore, the PDA coating serves as a polymer base, improving the adhesion of other bioactive cells and promoting osteogenesis through the sustained release of growth factors, such as BMP-2, which is crucial for regeneration [279].

Moreover, some biomaterials can also mimic bioelectrical characteristics specific to bone tissue, which has attracted the attention of numerous researchers. For example, endogenous electric fields in bone tissue have been shown to influence cell behavior by regulating cell proliferation and osteogenesis [280]. These advances have driven the design of novel piezoelectric materials, which can respond to mechanical and electrical stimuli by generating surface charges that promote cell adhesion and differentiation. This property in these materials occurs through a deformation of the net dipole moment induced by mechanical action [281]. The nanostructured composition of bone tissue is considered a piezoelectric material, which implies a mechanism that stimulates cells to respond to mechanical stress. In this sense, the most common materials, such as gallium nitride (GaN) and silicon carbide (SiC), have monocrystalline structures and lack a center of symmetry. They also respond to mechanical stress by generating electrical charges that promote bone formation, known as osteogenesis [282].

Additionally, nanopiezoceramic materials such as barium titanate (BT), boron nitride (BN), and zinc oxide (ZnO) have shown great promise in bone regeneration due to their piezoelectric and osteoinductive properties [283]:

- BT enhances the osteogenic differentiation of MSCs, promoting cell adhesion, proliferation, and migration.
- BN, in nanotube form, exhibits a high protein adsorption capacity, facilitating the osteogenic differentiation of MSCs.
- ZnO increases the bioactivity and mechanical strength of biomaterials, improving their integration with bone tissue.

These materials are incorporated into three-dimensional (3D) scaffolds to impart piezoelectric properties, enhancing bone formation. However, the biocompatibility of some of these materials may be limited at high doses, requiring optimization in their formulation.

Beyond piezoelectric materials, magnetic materials have also shown significant potential in bone regeneration, primarily through magnetic nanoparticles (MNPs). These nanoparticles exhibit superparamagnetic properties, meaning they do not retain magnetism without an external magnetic field, thereby minimizing the risk of local toxicity [284]. Examples of these nanoparticles include maghemite (Fe_2_O_3_) and magnetite (Fe_3_O_4_), both widely used in biomedicine due to their high magnetic susceptibility, allowing for precise functionalization control:

- Iron oxide nanoparticles have been shown to enhance osteoinduction in vitro, even without external magnetic stimulation;
- When incorporated into bioceramic or polymeric scaffolds, MNPs can further enhance bone regeneration through interactions with the physiological environment.

Combining magnetic biomaterials with external stimulation could open new avenues in advanced regenerative therapies. This would enable precise control over bone formation and enhance the effectiveness of treatments for complex bone defects [285].

### 4.1. Thermosensitive Materials in Promoting Bone Growth and Regeneration

Since temperature plays a crucial role in bone growth and regeneration, various thermal stimulation methods—such as direct, photothermal, and thermomagnetic approaches—have been reported to significantly influence this process [252]. Research has shown that thermal shock can activate Yes-associated protein (YAP), essential for bone growth [286]. Under thermal stress conditions, key proteins such as RUNX-2 and Col-1 exhibit increased expression, and YAP translocates to the nucleus, indicating its activation. Conversely, silencing YAP leads to opposite effects, highlighting its importance in the process [287]. Moreover, studies have demonstrated that YAP is a critical mediator in the osteogenic differentiation of ectomesenchymal stem cells, acting through transglutaminase 2 (TG2) [288]. Controlled temperature modulation, such as applying moderate localized heat (between 40 and 43 °C), could promote bone repair and growth [289].

Photothermal hydrogels have emerged as a promising tool in regenerative medicine due to their ability to convert light energy into heat, influencing cellular responses [290]. For example, Tan et al. (2022) demonstrated that these hydrogels can convert near-infrared (NIR) light into thermal energy, promoting the expression of heat shock proteins (HSP) and stimulating matrix metalloproteinase 2 (MMP2) activity as well as the ERK-Wnt/β–catenin–RUNX2 signaling axis. This process enhances osteogenic differentiation and new bone tissue formation in vivo under NIR conditions [291].

Additionally, there is growing interest in thermal methods for optimizing bone regeneration materials, such as bifunctional scaffolds based on graphene oxide (GO) modified with tricalcium phosphate (GO-TCP) [292]. Under NIR irradiation, this scaffold exhibits excellent photothermal properties, enhances osteogenic differentiation, and promotes the formation of new bone tissue compared to pure β-TCP scaffolds [292].

### 4.2. Piezoelectric Materials

Several materials exhibit piezoelectricity when mechanical deformation induces the formation of a net dipole moment, leading to their polarization. The biomedical application of such materials remains a novel area of research in regenerative medicine [280]. Bone itself is considered a nanostructured piezoelectric material, capable of generating a piezoelectric potential of 300 μV while walking [293]. This property has been proposed as a mechanism by which cells can detect and respond to mechanical stress [294]. In this context, the electrical stimulation of piezoelectric materials has attracted attention for its potential to enhance cell growth, repair, and differentiation, leading to the development of scaffolds with piezoelectric properties that promote the differentiation of mesenchymal stem cells (MSCs) into osteoblasts [295]. The physiological significance of bone mechanosensitivity has driven interest in researching inorganic piezoelectric ceramic materials, such as barium titanate (BT), boron nitride (BN), and zinc oxide (ZnO) [296]. While these materials exhibit a high piezoelectric coefficient, their biocompatibility may be limited at high doses, which could restrict their application in tissue engineering. However, these piezoceramics have demonstrated osteoinductive capabilities in vitro, supporting their use in bone regenerative biomaterials [297]. Incorporating these materials into 3D scaffolds can impart piezoelectric characteristics that facilitate bone formation. For instance, studies have shown that BT nanoparticles enhance the osteogenic differentiation of MSCs, and osteoblastic cells exhibit superior adhesion, proliferation, and migration within the pores of BT-based scaffolds. Similarly, boron nitride nanotubes (BNNTs) show a high protein adsorption capacity, enhancing MSC attachment, proliferation, and osteogenic differentiation [298].

Bone piezoelectricity can be enhanced by increasing the presence of negative charges in the physiological environment. These additional negative charges enhance osteoblastic function, promoting bone regeneration through increased mineralization at the site of the bone defect [293].

Polyvinylidene fluoride (PVDF) is a highly valued synthetic polymer in regenerative medicine due to its ability to induce electrical stimuli in bone defects. However, this material exhibits α, β, and γ crystalline structures, with only β and γ phases possessing excellent piezoelectric properties, while the α phase lacks this capability. This limitation arises from the challenges associated with selective phase separation [299,300]. Nonetheless, PVDF exhibits excellent mechanical properties, biocompatibility, and processability, enabling the development of tissue-specific functionalities. Additionally, PVDF copolymers such as poly(vinylidene fluoride-tetrafluoroethylene) (PVDF-TrFE), poly(vinylidene fluoride-chlorotrifluoroethylene) (PVDF-CTFE), and poly(vinylidene fluoride-hexafluoropropylene) (PVDF-HFP) demonstrate exceptional performance in biomedical applications. These materials combine thermal stability and chemical resistance with piezoelectric and ferroelectric properties, making them ideal for developing advanced medical devices, sensors, and actuators in tissue engineering and regenerative medicine [301].

Furthermore, studies have investigated the enhancement of PVDF nanofiber osteoconductivity and biocompatibility by incorporating graphene oxide (GO) into PVDF, referred to as PVDF-GO. This composite has been shown to stimulate the activity of ALP, osteocalcin, RunX2, and osteonectin genes, which is favorable for improving the piezoelectric properties of PVDF [302]. Table 6 summarizes some of the most promising materials in regenerative medicine due to their piezoelectric properties, which stimulate osteoblast differentiation and enable the creation of composites that enhance their piezoelectric and biocompatible properties.

### 4.3. Photosensitive Hydrogels in Bone Regeneration: Photoinitiators and Modification of Bioactive Properties

Photosensitive materials have emerged as a key tool in tissue engineering, particularly in synthesizing highly crosslinked hydrogels. In the presence of photoinitiators, these compounds form three-dimensional structures through photopolymerization, enabling their application in biomedicine and bone regeneration. Based on their activation mechanism, photoinitiators are classified into two categories:

- Type I generates free radicals via intramolecular bond cleavage under ultraviolet (UV) light. Representative examples include 2,2-dimethoxy-2-phenylacetophenone (DMPA) and lithium acylphosphinate (LAP).
- Type II, which requires lower activation energy and is more commonly used in biomedical applications, such as eosin Y and riboflavin [310,311]

One of the most significant advancements in this field is the modification of photosensitive hydrogels, such as methacryloyl gelatin (GelMA), by incorporating acrylamide and methacryloyl groups. These modifications enhance light responsiveness, improving crosslinking efficiency and structural stability. However, to maximize their regenerative potential, it is essential to incorporate bioactive agents such as drugs, nanoparticles, or bioactive ions capable of stimulating bone regeneration [312].

A significant development in applying photosensitive hydrogels in tissue engineering has been the introduction of GelMA enriched with black phosphorus nanosheets (BPN) [313]. This combination enhances the hydrogel’s mechanical properties and enables sustained phosphorus (P) release, a critical element in bone repair.

In vivo studies have validated the effectiveness of GelMA-BPN in bone regeneration, demonstrating improved integration with surrounding tissues. However, despite its advantages, critical challenges remain, including the following:

- Uncontrolled phosphate release, which may affect its osteogenic efficacy;
- Potential long-term cytotoxicity due to phosphorus accumulation in the cellular microenvironment.

Ongoing research into novel photoinitiators and structural modifications is expected to expand the applications of these hydrogels in bone regeneration by enhancing their biocompatibility and functionality. Furthermore, developing hybrid systems that combine GelMA with other innovative biomaterials could improve the efficiency and safety of these hydrogels for clinical applications.

### 4.4. Magnetically Responsive Hydrogels in Bone Regeneration: Nanoparticles, Stimulation, and Controlled Drug Release

The development of magnetically responsive hydrogels has introduced a novel approach in tissue engineering based on integrating magnetic nanoparticles (MNPs) within their structure. This combination enables the hydrogel to respond to an external magnetic field, regulates drug release, and influences cellular sensitivity, depending on the type and concentration of nanoparticles used [314].

MNPs incorporated into biomaterials can be categorized based on their composition, with the most notable types being the following:

- Iron oxides, such as magnetite (Fe_3_O_4_) and maghemite (γ-Fe_2_O_3_), are widely used due to their biocompatibility and magnetic properties;
- Metallic nanoparticles, composed of cobalt, iron, and nickel, which exhibit higher magnetization but also pose increased cytotoxicity risks.

Iron oxide nanoparticles (MNPs) have been particularly successful in biomedical applications, especially bone regeneration, due to their ability to induce osteogenic signaling in combination with magnetic stimulation [282]. However, MNP use must be carefully regulated, as excessive iron exposure can trigger the production of reactive oxygen species (ROS), increasing the risk of cellular toxicity.

Magnetic nanoparticles can be synthesized through various methods, including the following:

- High-intensity ultrasound, which facilitates the rapid and uniform formation of nanoparticles;
- Thermal decomposition, a technique used to produce MNPs with controlled size and morphology;
- Co-precipitation, a widely adopted method due to its simplicity and high efficiency in generating superparamagnetic nanoparticles [315].

Recent studies have shown that magnetic stimulation of hydrogels loaded with magnetic nanoparticles (MNPs) significantly enhances bone tissue regeneration. These biomaterials allow controlled drug release under magnetic influence and dynamically adapt to physiological changes, making them a promising strategy in regenerative medicine [316].

The ongoing exploration and optimization of magnetically responsive hydrogels continue to expand possibilities for advanced regenerative therapies, enhancing treatment effectiveness for complex bone defects. Combining these materials with osteogenic growth factors or mesenchymal stem cells (MSCs) could further strengthen scaffold bioactivity, offering more effective solutions for bone regeneration and advanced biomedical applications [316].

## 5. The Future: Advances in Gene Therapies Targeting Tissue Engineering to Promote Bone Formation

In recent decades, gene therapy has gained widespread popularity in various clinical applications, including various bone defects. In this regard, several preclinical investigations have demonstrated that cell and gene therapies can be enhanced using biomaterials such as scaffolds with specific structural characteristics that allow the localized and controlled release of various genetic products. This enhances the effect of local treatments and reduces systemic side effects and the regulatory capacity of various endogenous genes and proteins [317].

These combined methods have developed so-called gene-activated materials (GAMs), which involve templates doped with vectors carrying therapeutic genes. This is attractive because they can transfect recipient cells and stimulate the local production of essential proteins and molecules involved in bone regeneration [318]. However, this approach has a significant limitation in its application in bone defects caused by disease. It depends on the number of healthy cells available for transfection at the affected site, which can be difficult in tissues severely affected by diseases such as osteoporosis. Given this situation, gene-activated tissue implants in combination with cell therapies are feasible [318]. An attractive approach to delivering GAMs to specific sites of severe bone defects, which takes place through viral and non-viral delivery vectors, is adenovirus (AdV), which is widely used in clinical trials. An advantageous feature of AdV is that the transported genes are expressed independently outside the genome of the host cells [319].

A successful case was reported by Sharma et al., who used AdV with morphogenic proteins to express vascular endothelial growth factor (VEGFA) in mesenchymal stem cells. Subsequently, the edited mesenchymal stem cells were implanted into mesoporous scaffolds, favoring bone marker expression and new bone formation [320]. Adeno-associated viruses (AAVs) are also frequently employed in clinical trials. It is of great interest due to its non-pathogenicity and ability to infect various cells. For example, Oh et al. successfully used AAVs loaded with peptide motifs targeting an allograft in patients with osteoporosis to transduce mesenchymal stem cells present in the graft, significantly improving bone density [321]. Furthermore, lipid-based systems have gained popularity for delivering gene material in clinical applications. For example, liposomes are attractive for the delivery of gene material due to their ability to integrate with cell membranes. This approach has silenced genes implicated in bone integrity-related diseases [322].

Other interesting approaches for the controlled and localized release of genetic material consist of using polymeric biomaterials, among which the most notable are chitosan polymers, PEI, dendritic macromolecules, and polyphosphoesters [323]. These release systems present significant improvements, such as a greater transfection capacity and slowing down DNA degradation. In addition, their structural characteristics can be adapted to improve their compatibility with the physiological microenvironment [324,325].

Systems based on inorganic nanoparticles also represent a significant advance in the transport of drugs and bioactive molecules across membranes. Among the most common nanoparticles for the transport and release of molecules are silica and calcium phosphate nanoparticles [326]. Other nanoparticles, such as gold, are highly efficient because they can successfully couple to interfering RNA (RNAi), generating interference in the expression of cathepsin K (an enzyme associated with bone resorption) in osteoblastic cells, optimizing angiogenesis [327].

However, a problem must be addressed to ensure greater efficiency of the techniques and methods mentioned in the previous sections. For example, although several studies have incorporated growth factors such as fibroblast growth factors (FGFs), transforming growth factors (TGFs), platelet-derived growth factors (PDGFs), angiopoietins (Angs), and in some cases interleukins that promote vascularization in severe and extensive bone defects [328], it is common for these procedures to occur under hypoxic conditions, which negatively affects local cells that are essential for bone tissue repair, especially bone defects that involve loss of bone density, generating morbidity in patients. Given this situation, biomaterials must guarantee good vascularization during recovery [328]. In this regard, future research should ensure vascularization after orthopedic procedures in severe fractures to avoid morbidity in patients.

## 6. Conclusions and Future Perspectives

Advances in regenerative medicine to treat bone defects have undergone unprecedented evolution. This suggests a positive advance for orthopedic methods, from traditional approaches to more sophisticated methods using biomaterials to treat various bone defects. This study determined that although autologous and allogeneic bone are significant and commonly used in treating bone defects, they face serious challenges that must be addressed. For example, in orthopedic treatments using autologous bone, the additional surgical procedures required increase the risk of complications and morbidity in patients. Additionally, the use of allogeneic bone also presents limitations, including a high risk of immune rejection and serious infections that can compromise the patient’s well-being. These limitations and challenges have prompted the development of novel materials designed to mimic bone’s chemical composition and microenvironment, promoting its integration.

In this regard, the various advances in the design and synthesis of nanobiocomposites and nanostructured biomaterials have enabled breakthroughs in treating bone defects. For example, applying hydrogels responsive to external stimuli and controlled-release systems for drugs and growth factors promoting osteogenesis represents a crucial advance, enabling bone tissue regeneration. Thermoresponsive or pH-dependent hydrogels exhibit exceptional capabilities in the controlled release of bioactive compounds, promoting bone regeneration. However, significant challenges remain related to the biodegradability of some potential materials, which can hinder or interfere with the healing process.

On the other hand, nanobiocomposites based on hydroxyapatite and biodegradable polymers such as polylactic acid (PLA) have been demonstrated to enhance the biocompatibility and mechanical properties of the material significantly. However, the high costs associated with large-scale production and the need to enable availability in clinical settings and regulatory approval represent a prohibitive aspect of the widespread implementation of these materials.

Furthermore, an appropriate structure–activity relationship is a crucial factor in the efficiency of these materials, as their osteogenic potential largely depends on their ability to mimic the extracellular matrix and promote osteoconduction and osteoinduction. Ceramics with piezoelectric properties, such as barium titanate (BT) and zinc oxide (ZnO), demonstrate good cell stimulation properties due to their piezoelectric properties. However, their structures must be optimized to maximize their electrical activity without compromising bioactivity.

Similarly, doping these biomaterials with magnetic nanoparticles has enhanced their osteogenesis-inducing properties. Since these particles are integrated into bioceramic or polymeric scaffolds, this doping can significantly improve bone tissue regeneration through external magnetic stimulation. Further research is essential to overcome these limitations, minimize the risks of toxicity and rejection, and improve the uniform dispersion of nanoparticles within these biomaterials.

It has been demonstrated that current biomaterials provide effective solutions for treating bone defects. Their success in orthopedic applications is strongly influenced by the type of chemical optimization and their bioactive properties, as well as overcoming the challenges associated with scalability, biocompatibility, and regulatory approval. Future research should focus on the following goals:

1. Design and synthesize hybrid materials that integrate and enhance osteoinductive, osteoconductive, and antimicrobial properties.
2. Optimize the thermal stability and biocompatibility of everyday materials used in orthopedic procedures to synchronize the biodegradation time with the healing of the bone defect.
3. Explore innovative, low-cost functionalization strategies using nanoparticles that reduce their cytotoxic potential and enhance their integration into common biomaterials in orthopedic procedures.
4. It is necessary to strengthen the scalability of these materials to ensure the effective transition and widespread use of these materials in clinical procedures.

Continued progress in this field will ensure the effectiveness of various personalized treatments according to the type of bone defect. This is essential to guarantee the treatment’s effectiveness and ensure patients’ integrity, improving their quality of life.

Furthermore, ethical and legal approval for the use of these materials is an important aspect that must be addressed. That is, existing regulations must be strict to ensure safety and rapid recovery, ensuring a better quality of life for patients, but certain flexibilities must be maintained in some aspects. For example, in ethical terms, the approval and informed consent of patients with serious bone defects and diseases that make treatment difficult and require more sophisticated procedures, such as cell transfection or localized delivery of genetic material, is essential. This aspect is important because it ensures that patients understand the risks and benefits of this type of treatment, as reported Pal et. al. [329]. In addition, the development of more effective techniques and methods that ensure their reliability, complying with the appropriate selection and sterilization protocols, is necessary. Likewise, from a legal point of view, the regulations associated with these procedures must include stricter regulations on the origin of the tissues, as well as compatibility tests and biocompatibility measures that allow minimizing the risk of rejection and/or infections, guaranteeing patient safety [330,331].

## Figures and Tables

**Table 1:** Classification of the type of bone fractures and their main clinical characteristics.

Classification	Type of Fracture	Description	Graft or Biomaterial Requirements	Ref.
According to the type of fracture	Cross	Line that forms a right angle with the longitudinal axis of the bone.	-	[39]
	Oblique	The line is presented at an angle about the longitudinal axis of the bone.	-	
	Spiral	A helix-shaped line that surrounds the contour of the bone.	-	
	Linear	Line that extends parallel to the longitudinal axis of the bone without displacement.	-	
	Green stem	A partial fracture is common in childhood, where the bone is deformed by bending without breaking completely.	Hydroxyapatite to mimic the chemical composition of bone and promote cell migration and/or communication at both ends of the fracture.	
According to the type of damage	Complete	The bone fractures into two or more segments.	Phosphate-based nanobiocomposites to promote bone regeneration in complicated fractures.	[40]
	Incomplete	Cracks or incomplete fractures in the bone.	-	
	Comminuted	The bone breaks into several fragments.	Grafts extracted from the same patient (autologous grafts) or a donor (allogeneic grafts).	
	Composed	The fracture pierces the skin, exposing the bone to the outside environment.	Synthetic biomaterials such as hydroxyapatite and/or calcium phosphates are required as fillers in bone defects.	
	Closed	There is no connection between the fracture and the external environment.	Nanobiocomposites, hydroxyapatites, and 3D scaffolds with stem cells to promote osteogenesis.	
According to the mechanism of injury	Flexion	It occurs as a result of forces that induce the bone to bend.	-	[41]
	Torsion	It arises from forces that cause a twisting movement.	-	
	Compression	It occurs due to forces that exert pressure on the bone, causing it to collapse.	-	
	Sharing	It occurs when two forces exert pressure on the same bone in opposite directions.	-	
	Pathological	They occur due to medical conditions compromising bone strength, such as osteoporosis or tumors.	Osteoinductive materials that stimulate bone formation.	
	Fatigue or stress	Minor fractures caused by repeated stress are common among athletes.	Nanostructured grafts to improve the stability and mechanical resistance of the affected area.	
According to the energy of trauma	High energy	Caused by severe trauma, such as in accidents, these fractures often show considerable fragmentation and affect the surrounding tissues.	-	[42]
	Low energy	They occur due to falls or inappropriate movements, especially in older people with fragile bones.	Hydroxyapatites, calcium phosphates, 3D scaffolds with stem cells and growth factors to promote osteogenesis and to treat compromised bone mass.	

**Table 2:** Preclinical studies in animals and humans, scaffold modifications, the sterilization process, and the implantation site.

Type of Material	Material	Manufacturing Technique	Modifications	Type of Sterilization	In Vivo Applications	Implantation Site	Ref.
Ceramics	α-TCP ^1^	Inkjet printing.	-	Autoclave	Human	Maxillofacial	[70]
	TCP ^2^	Dispensing by tracing.	Impregnation with osteoblasts and coating with collagen.	Plasma	Sheep	Calvary	[71]
	β-TCP ^3^	Robocasting.	Coating with mesoporous bioglasses.	-	Rabbit	Mandible, skull	[72,73]
	Dicalcium phosphate	Dispensing by tracing.	-	γ-irradiation	Goat	Lumbar area	[74]
	Biphasic calcium phosphates	Robocasting.	Impregnation with bone morphogenetic proteins.	Heating	Pig and rabbit	Mandible, tibia	[75]
Composites	PCL ^4^/PLGA ^5^	Additive manufacturing.	Collagen enriched with rhBMP-2.	Ultraviolet	Rabbit	Calvaria and radio	[76,77]
	PCL/PLGA/β-TCP	Additive manufacturing.	Collagen enriched with rhBMP-2.	-	Rabbit	Calvaria	[76,78]
	PLGA/β-TCP	Fused deposition modeling.	HA coating.	Ethylene oxide	Rabbit	Femur	[79]
	PCL/HA ^6^	Selective laser sintering.	-	-	Rabbit	Femur	[80]
	PLA/nHA ^7^	Fused deposition modeling.	-	Impregnation with ethanol	Rabbit	Femur	[81]
	TCP/CS ^8^/ Collagen hydrogel	Dispensing by tracing.	Impregnation with osteoblasts.	Plasma	Sheep	Calvaria	[71]
Synthetic biodegradable polymers	PCL	Selective laser sintering.	Impregnation with recombinant human platelet-derived growth factors.	Ethylene oxide	Human	Periodontal	[82]
	PLA ^9^	Fused deposition modeling.	-	Impregnation with ethanol	Rats	Femur	[83]
	PLGA	Dispensing by tracing.	-		Rabbit	Iliac bone	[84]
Non-degradable synthetic polymers	PEKK ^10^	Selective laser sintering.	Autologous bone.	Autoclave	Sheep	Calvaria	[85]
		Fused deposition modeling.	-	-	Human	Rib	[86]

^1^: α-tricalcium phosphate. ^2^: Tricalcium phosphate. ^3^: β-tricalcium phosphate. ^4^: Polycaprolactone. ^5^: poly(lactic-co-glycolic acid). ^6^: Hydroxyapatite. ^7^: nanohydroxyapatite. ^8^: Chitosan. ^9^: Polylactic acid. ^10^: polyether ketonetone.

**Table 3:** Types of bone grafts: main characteristics, origin, advantages, and disadvantages.

Type of Bone Graft	Origin	Advantages	Disadvantages	Reported Cases	Ref.
Autologous bone	It is extracted from the same patient.	It presents functional osteoblasts, excellent osteoconductivity, biocompatibility, and bone induction properties.	It requires additional surgical procedures, which may compromise nerves, tissues, and arteries and increase morbidity.	Autologous iliac bone graft for the treatment of tibial nonunion. This graft has demonstrated osteoblast activity and BMP- and glycoprotein-induced osteogenesis in 51 patients.	[116]
Allogenic bone	The graft comes from a donor. It may be genetically different from the donor, but from the same species as the recipient.	It presents functional growth factors, various types of functional tissue cells, extracellular matrix, and other relevant factors.	It presents a high antigenic risk, the spread of diseases, and the risk of rejection.	Allogeneic bone screw implantation in hands and feet in 32 patients. The implant demonstrated rapid immune acceptance by the patient and a swift recovery with minimal pain.	[117]
Xenogeneic bone	The graft originates from a species different from the patient’s (usually bovine or porcine), thus having a distinct genetic origin.	They have a large volume and are abundantly available. Additionally, some species exhibit good osteoconductivity.	It is highly antigenic, has a high propensity for disease, and presents risks of rejection by the patient.	In 11 patients, a xenogeneic bone ring graft was used to treat horizontal alveolar bone defects. All patients achieved 100% implant survival and acceptance rates.	[118]

**Table 4:** Classification of some ceramic materials and their applications in regenerative medicine.

Generation of the Material	Material Name	Biological Behavior	Clinical Application	Example	Ref.
First generation	Aluminas, Zirconia	Bioinert	Coatings for tissue growth. Orthopedic, dental, and maxillofacial applications.	ZrO_2_, composed of PE-HA y Al_2_O_3_	[167]
	Titanium Nitride, Zirconium Nitride	Bioinert	Knee prosthesis, anti-wear coating on bone joints.	TiN, ZrN	[168]
	Silicon Nitride	Bioinert	Anti-wear coating on joints.	Si_3_N_4_	[169]
Second generation	HA	Bioactive	Bone cavity filling, cartilage implants, vertebral replacement, hip implants, bone and orthopedic scaffolding.	Carbon fiber composites-PLA, HA, bioactive glass, and glass ceramics	[170]
	Bioglass	Bioactive	Bone replacement.	Bioactive glass and glass-ceramics	[171]
	β-TCP	Bioactive O/Biodegradable	Bone replacement.	Ca_3_(PO_4_)_2_	[172]
	HA/PCL	Biodegradable	Scaffolds in tissue engineering.	-	
	DCPD	Biodegradable	Bone regeneration, osteoconductivity.	-	[173]
	Calcium Phosphate		Promotes tissue growth and vascularization.	Ca_3_(PO_4_)_2_	[174]
Third generation	Biodegradable ceramics	Bioactive/Biodegradable	These materials are based on scaffolds that support cell attachment and growth, actively participating in the healing process by releasing growth factors or other bioactive substances.	-	[175]

**Table 5:** Examples of polymer and nanobioglass combinations for bone regeneration in regenerative medicine.

Biopolymers	Nanobioglass	Resulting Compound	Applications	Ref.
Alginate	Mesoporous with 10% by weight.	Microspheres	Increases drug loading and release capacity due to its surface area.	[193]
PHBV	45S5 10% by weight.	Scaffolding	Cartilage transplants and repair.	[194]
PCL	Mesoporous 5% by weight.	Scaffolding	Bone regeneration.	[195]
PLLA	Mesoporous 15% by weight.	Scaffolding bases	Bone regeneration.	[196]
PLGA	45S5	Microspheres	Endothelial activity	[197]
Collagen	Mesoporous 10% by weight.	-	Cartilage regeneration and transplantation.	[198]
Chitosan	-	Nanofibers	It improves biomineralization and stimulates the formation of bone extracellular matrix by osteoblasts.	[199]
PLA	Mesoporous 10% by weight.	Nanocomposite	Repairs bone tissue and prevents microbial contamination.	[200]
CS-G-NBG-GO	-	Scaffold	It has excellent cytocompatibility with MG-63 cell lines.	[201]

PHBV: Poly(3-hydroxybutyrate-co-3-hydroxyvalerate). PLLA: Poly-l-lactic acid. PLGA: Poly(lactic-co-glycolic acid). PLA: Polylactic acid.

**Table 6:** Piezoelectric materials used in regenerative medicine.

Material	Cells	Method	Advantages	Disadvantages	Ref.
LNKN	Osteoblasts	CIP ^1^	It promotes extracellular matrix-like topography, improves biological performance, and enhances piezoelectricity through CIP using sodium, lithium, and potassium niobate samples. It also exhibits positive in vitro effects on osteoblast adhesion and proliferation.	It is limited in permissible doses, has a long synthesis time, and is expensive, which restricts its applicability in regenerative medicine. Furthermore, these improved properties only occur through unconventional methods such as CIP.	[303]
LNKN	-	Foam formation	It exhibits relative dielectric and piezoelectric constants of 67.29 and 48 pC/N, respectively. The piezoelectric properties are optimized by 65% by weight of solids and 30% by weight of foaming agent, which improves its ability to stimulate osteoblast production and facilitates its application and production.	Increasing the amount of foaming agents increases the material’s porosity, resulting in a more uniform pore structure. However, this decreases the density and dielectric constant.	[304]
KNN ^2^	BMSC ^3^	Mechanosynthesis	Improved cell adhesion and colony reduction in *Staphylococcus aureus* bacteria, which is attractive for preventing infections, and BMSC cell proliferation induced by piezoceramics.	Low efficiency and high costs.	[305]
KNN/HA	MG-63	Mechanosynthesis	The mechanical properties of the HA composite containing 30% by weight of KNN are as follows: a hardness of 93%, a fracture toughness of 209%, a flexural strength of 88%, and a compressive strength of 112%. This is attractive for applications in problematic bone defects.	A significant reduction in bacterial viability (*E. coli* and *S. aureus*) was observed in samples of HA-KNN composites subjected to in vitro polarization, which is concerning for applications in regenerative medicine, particularly bone regeneration and limb preservation.	[306]
Barium strontium titanate (BST)/β-TCP	BMSC	3D printing	Samples containing 40% β-TCP and 60% BST exhibit high compressive strength, flexural strength, elasticity, and a high Young’s modulus. Furthermore, these samples exhibit significant bone apatite formation after 28 days in SBF.	It involves temperatures of up to 770 °C, where significant weight loss is recorded due to solvent evaporation during drying. This situation limits its performance and increases costs.	[307]
PHB ^4^/PCL	Osteoblasts	Electrospinning	Improved wettability and cell dispersion in PHB (piezoelectric) and PCL (non-piezoelectric) scaffolds. This combination improves mechanical strength and biocompatibility.	Reduction in the piezoelectric coefficient d33 (from 2.5 ± 0.3 to 2.1 ± 0.4 pC/N) and in the surface electric potential (from 510 ± 56 to 458 ± 25 mV) by surface treatment with diazonium.	[308]
PHB/CaCO_3_ PHBV/CaCO_3_	Osteoblasts	Ultrasound	The piezoelectric coefficient of PHB is higher (d33 = 3.0 ± 0.5 pC/N) than PHBV scaffolds (d33 = 0.7 ± 0.5 pC/N). Furthermore, PHB exhibits higher porosity (approximately 15%) than PHBV scaffolds due to the homogeneous growth of CaCO_3_ on the 3D fibrous structures.	Low efficiency.	[309]

^1^ Cold isostatic pressing. ^2^ Potassium niobate. ^3^ Bone marrow stromal cells. ^4^ Polyhydroxybutyrate.

## Data Availability

The data presented in this study are openly available in the article.
